# A New Projection From the Deep Cerebellar Nuclei to the Hippocampus *via* the Ventrolateral and Laterodorsal Thalamus in Mice

**DOI:** 10.3389/fncir.2019.00051

**Published:** 2019-08-09

**Authors:** Pauline Bohne, Martin K. Schwarz, Stefan Herlitze, Melanie D. Mark

**Affiliations:** ^1^Department of General Zoology and Neurobiology, Ruhr-University Bochum, Bochum, Germany; ^2^Institute of Experimental Epileptology and Cognition Research (EECR), University of Bonn Medical School, Bonn, Germany

**Keywords:** cerebellar-hippocampal projection, cerebellum, hippocampus, thalamus, circuitry, rabies, rAAV

## Abstract

The cerebellar involvement in cognitive functions such as attention, language, working memory, emotion, goal-directed behavior and spatial navigation is constantly growing. However, an exact connectivity map between the hippocampus and cerebellum in mice is still unknown. Here, we conducted a tracing study to identify the sequence of transsynaptic, cerebellar-hippocampal connections in the mouse brain using combinations of Recombinant adeno-associated virus (rAAV) and pseudotyped deletion-mutant rabies (RABV) viruses. Stereotaxic injection of a primarily anterograde rAAV-WGA (wheat germ agglutinin)-Cre tracer virus in the deep cerebellar nuclei (DCN) of a Cre-dependent tdTomato reporter mouse resulted in strong tdTomato labeling in hippocampal CA1 neurons, retrosplenial cortex (RSC), rhinal cortex (RC) as well as thalamic and cerebellar areas. Whereas hippocampal injections with the retrograde tracer virus rAAV-TTC (tetanus toxin C fragment)-eGFP, displayed eGFP positive cells in the rhinal cortex and subiculum. To determine the sequence of mono-transsynaptic connections between the cerebellum and hippocampus, we used the retrograde tracer RABVΔG-eGFP(EnvA). The tracing revealed a direct connection from the dentate gyrus (DG) in the hippocampus to the RSC, RC and subiculum (S), which are monosynaptically connected to thalamic laterodorsal and ventrolateral areas. These thalamic nuclei are directly connected to cerebellar fastigial (FN), interposed (IntP) and lateral (Lat) nuclei, discovering a new projection route from the fastigial to the laterodorsal thalamic nucleus in the mouse brain. Collectively, our findings suggest a new cerebellar-hippocampal connection *via* the laterodorsal and ventrolateral thalamus to RSC, RC and S. These results strengthen the notion of the cerebellum’s involvement in cognitive functions such as spatial navigation *via* a polysynaptic circuitry.

## Introduction

The cerebellum was exclusively associated with motor coordination related tasks such as balance, precise timing of movements or motor learning. However, recent functional brain imaging studies with cerebellar degenerative disease and cerebellar lesioned patients support the cerebellar contribution in cognitive functions such as attention, language, working memory, emotion, and in visuospatial navigation (Schmahmann and Pandya, 1991; Timmann and Daum, 2007; Baillieux et al., 2008a,b; Molinari et al., 2008; Timmann et al., 2010). In support of these human studies, rodents with impairments in their cerebellum demonstrated a reduction in hippocampal based behavioral tasks such as goal-directed and spatial navigation tests (Colombel et al., 2004; Burguière et al., 2010; Rochefort et al., 2011). An anatomical cerebellar-hippocampal connection in the mouse brain supporting its participation in spatial navigation has not been investigated. It is not yet clear whether this is through a direct monosynaptic projection from the cerebellum to the hippocampus, or by polysynaptic transmission involving e.g., the thalamus (Rochefort et al., 2013). Moreover, the exact sequence and the identity of connected neuronal populations are not known.

Evidence for a direct, monosynaptic connection between the cerebellum and hippocampus is weak. In the 1980s transient direct projection from the cerebral cortex to the deep cerebellar nuclei (DCN) and cortex in young kittens, rabbit fetuses and in pouch young North American opossum have been reported (Tolbert and Panneton, 1983, 1984; Cabana and Martin, 1986; Tolbert, 1989a,b). Additionally, a sparse projection from the cerebellum to the neocortex in adult rats was demonstrated (Wild and Williams, 2000). Direct cerebrocerebellar projections have also been reported in chicken and zebra finches, however, they are sparse and temporary (Wild and Williams, 2000; Liu et al., 2012). Since most of these studies used polysynaptic radiolabeled amino acids or wheat germ agglutinin conjugated to horseradish peroxidase (WGA-HRP) as tracers, the interpretation of these results is difficult.

To explore the neuronal connectivity between structures in the mammalian brain, recombinant viruses including AAV and/or modified rabies viruses have presently become the tool of choice. Recombinant adeno-associated viruses (rAAVs) offer great advantages in cell-specific labeling due to deletion of almost all coding sequences resulting in non-pathogenicity, loss of self-reproduction (Xiao et al., 1997; Büning et al., 2008; Kwon and Schaffer, 2008) and long-term expression of introduced proteins combined with little to no mammalian immune reaction (Kaplitt et al., 1994; Xiao et al., 1996, 1997; Chamberlin et al., 1998). The main disadvantage of rAAVs in tracing studies is their incapability to cross synaptic junctions, although certain rAAV serotypes have been reported to support anterograde transsynaptic transport at high titers (Ohta et al., 2011; Deneris and Wyler, 2012). Yet, they can express proteins that cross synapses (WGA), however, this strategy can not distinguish between strong polysynaptic from potential weak direct connections (Wickersham et al., 2007a). The sequence of traversed synapses can only be roughly estimated. Thus, only an additional monosynaptically restricted tracing approach can unequivocally determine the sequence of synaptic connections. Therefore, we confirmed our rAAV tracing results with tracings utilizing a deletion-mutant rabies virus RABVΔG-eGFP (EnVA). This modified rabies virus (RABV) expresses eGFP at the expense of the rabies virus glycoprotein, limiting its potential for retrograde transsynaptic transport. To reveal monosynaptic connectivity maps the glycoprotein has to be transcomplemented in the initially infected cell population (Ugolini, 1995; Kelly and Strick, 2000; Wickersham et al., 2007a,b). This transcomplementation can be accomplished upon rAAV targeted glycoprotein expression in the source cell population (Niedworok et al., 2012). Therefore this tracing method can determine the hierarchy of anatomical connectivity in the brain and a potential cerebellar-hippocampal monosynaptic connection.

Here, we report a tracing study, identifying a sequential connectivity map between the cerebellum and the hippocampus in the mouse brain. Stereotaxic injections with the tracer virus rAAV CMV-WGA-Cre in the DCN of tdTomato-reporter mice resulted in stained neurons in the rhinal cortex, subiculum, hippocampal CA1 region and also to some extent in the thalamus. In contrast, injections of the same virus in the hippocampus resulted in fluorescently stained Purkinje cells and molecular layer interneurons, in addition to stainings in the pons, thalamus and hippocampus, including CA1 pyramidal neurons, neurons of the dentate gyrus (DG), pre- and parasubiculum and lateral entorhinal cortex. However, after injection of the retrograde tracers rAAV TTC-eGFP in the DG and rAAV CMV-WGA-Cre in a tdTomatoJ reporter mouse in cerebellar CrusI/CrusII region, we detected overlapping fluorescence in rhinal cortex (RhC), DG and subiculum (S), indicating that at least the same areas are involved in forming a cerebellar-hippocampal connection. To finally determine the hierarchy of monosynaptic connections between the cerebellum and hippocampus, we applied a modified retrograde RABV, SADΔG-eGFP (EnVA) in the rhinal cortex (RhC), subiculum (S), DG and retrosplenial cortex (RSC). We found monosynaptic projections from the laterodorsal and ventrolateral thalamus to the S and retrosplenial agranular cortex (RSA), of which both are reported to be involved in spatial navigation (Rochefort et al., 2013). eGFP positive neurons were detected in mainly the contralateral interpositus and fastigial, but not dentate nucleus of cerebellar DCN. Taken together, our findings suggest a new projection from the fastigial nucleus to the laterodorsal thalamic nuclei to S and RSC, which are connected to the hippocampus. In addition, our results show a potential sequence of polysynaptic cerebellar-hippocampal connections *via* the thalamus to various cortical areas.

## Materials and Methods

### Plasmid Construction

pAAV constructs (pAAV-CMV-WGA-CRE and pAAV-CMV-TTC-GFP) were amplified by PCR from the original vectors and cloned into the pAAV-MCS (Stratagene). WGA-Cre was amplified from pAAV-EF1a-mCherry-IRES-WGA-Cre (University of North Carolina Vector Core, Chapel Hill, NC, USA). TTC was amplified from psK1-TTC, which was kindly provided as a gift by Dr. Neil F. Fairweather (Imperial College London, UK).

### Virus Production

rAAV8 production of virus from pAAV-CMV-WGA-Cre and pAAV-CMV-TTC-eGFP constructs were performed by a modified method (Grieger et al., 2006). Briefly, low passage 293T cells were cotransfected with pAAV-CMV-WGA-Cre or pAAV-CMV-TTC-eGFP, pAAV-RC, and pHelper using the Polyethylenimine (PEI) based protocol. Three days after transfection cells were removed from the dishes, pelleted (3,700 g, 20 min, 4°C), resuspended in 10 ml lysis buffer (150 mN NaCl, 50 mM Tris-HCl, pH 8.5) and lysed *via* six freeze/thaw cycles in dry ice/ethanol and 37°C water bath (each 15 min). To get rid of free DNA, cell suspension was treated with DNase I (Roche) for 30 min at 37°C. The cell debris was spun down at 3,700 g for 20 min at 4°C. The supernatant was collected in a syringe and filtered into a 15 ml falcon tube through a 0.2 μm filter to obtain the crude lysate. Then the supernatant was resuspended in a polyethylene glycol (PEG) solution overnight at 4°C and pelleted at 3,700 g for 20 min at 4°C. The pellet was resuspended in PBS, 0.001% pluronic and aliquots were stored at −80°C until further use.

SADΔG-eGFP (EnVA) and helper plasmids pAAV8-CBA-mRFP-IRES-TvA and pAAV8-CBA-RG-mCherry were produced as previously described (Niedworok et al., 2012). Briefly, BHK cells were plated at a density of 1.5 × 10^7^. The following day, cells were transfected with 15 mg plasmid pCAGG/SAD-G by CaP transfection. Twenty-four hours later rabies virus SADΔG-eGFP was added at a multiplicity of infection (MOI) of 3. Forty-eight hours later the SADΔG-eGFP containing supernatant was equally distributed into four 15 cm plates containing pCAGGs/SAD-G (15 mg/plate) transfected BHK cells (1.5 × 10^7^ cells/plate). Two days later the virus-containing supernatant was applied onto four 15 cm plates containing BHK-EnvARGCD cells (~1.5 × 10^7^ cells/plate) at a MOI of 1.5 for pseudotyping. Twelve hours later cells were trypsinized and replated onto eight 15 cm dishes. Pseudotyped rabies virus-containing supernatant was harvested 2 days later. The supernatant was spun at 2,000 rpm at 4°C for 10 min. and subsequently filtered through a 0.45 mm filter (Nalgene SFCA Bottletop Filter, Thermo Fisher Scientific, Waltham, MA, USA). The filtered virus suspension was centrifuged for 90 min at 25,000 rpm (SW28, 4°C) in a Beckmann 80 K ultracentrifuge (Beckman Coulter, Brea, CA, USA). After centrifugation the supernatant was discarded and the pellet was aspirated in ice-cold PBS (pH 7.4). Pseudotyped rabies virus-containing solution was aliquoted in 6 μl aliquots and frozen at −80°C.

### Mice

For the tracing study adult male and female mice obtained from JAX labs, C57Bl6/J (JAX 000664) and Gt(ROSA)26Sor^tm9(CAG-tdTomato)Hze^/J (abbreviated as tdTomato^+/+^; JAX 007909, Madisen et al., 2010), were used. Mice were housed in a 12 h light/dark cycle with food and water *ad libitum*. The present study was carried out in accordance with the European Communities Council Directive of 2010 (2010/63/EU) for care of laboratory animals and approved by a local ethics committee (Bezirksamt Arnsberg) and the animal care committee of North Rhine-Westphalia, Germany, based at the LANUV (Landesamt für Umweltschutz, Naturschutz und Verbraucherschutz, Nordrhein-Westfalen, D-45659 Recklinghausen, Germany). The study was supervised by the animal welfare commission of the Ruhr-University Bochum. All efforts were made to minimize the number of mice used for this study.

### Intracranial Injections

Viruses were injected in adult mouse brains for each specified region tested. Mice were deeply anesthetized with 1.5%–2.0% isoflurane and placed into a stereotactic frame (Narishige, Japan). The skin was opened with a sagittal incision along the midline. A small craniotomy was performed for virus injections. 0.2–1 μl of viruses were applied in 100 μm steps using pressure injection in 2 min intervals (see Table 1). A customized glass pipette attached to a 5 ml syringe was used for virus delivery. At the end of injection the skin was sutured (Surgicryl Monofilament, Belgium). After the surgery, animals received subcutaneous injection of carprofen (2 mg/kg) for analgesia. Animals were placed individually into their home cages to recover.

**Table 1:** Injection site coordinates, number of animals and injected volumes of rAAV8-CMV-WGA-Cre and rAAV8-CMV-TTC-eGFP tracers.

	Structure	AP* (mm)	ML* (mm)	DV* (mm)	*n*	Volume rAAV (μl)
Hippocampus	Dentate gyrus	−2.54	± 1.5	1.8–1.6	3	1
	CA1/CA3	−2.18	± 2.1	1.9–1.5	2	1
Cingulate cortex	Agranular and granular RSC	−2.54	± 0.3	0.75–0.25	3	1
Cerebellum	Medial (fastigial) nucleus	−6.25	± 0.7	2.4–2.2	3	1
	Crus I/II	−6.4	−2.7	1.7–1.07	2	1

*AP, anterior-posterior; ML, mediolateral; DV, dorsoventral (all relative to Bregma).

rAAV8-CMV-WGA-CRE was injected into the fastigial nucleus of the DCN, CrusI/CrusII of the cerebellar cortex and DG, AAV8-CMV-TTC-eGFP in the hippocampus proper (CA1/CA3) and DG of Gt(ROSA)^26Sortm9(CAG-tdTomato)Hze^/J mice. Coordinates and volumes of injected viruses are listed in Table 1. Expression times varied between 3 and 8 months.

The helper viruses for rabies infection (rAAV8-CBA-mRFP-IRES-TvA and rAAV8-CBA-RG-mCherry, ratio 1:2) were injected 1 week before the deletion mutant rabies virus RABVΔG-eGFP to allow stable infection and expression of TVA and RG. Rabies virus was injected into the hippocampus, RSC, rhinal cortex, laterodorsal and ventrolateral thalamus of C57BL6/J mice. For injected volumes and injection sites, see Table 2. Mice were perfused and analyzed 7 days after RABV application.

**Table 2:** Injection site coordinates and number of animals and injected volumes of rAAV8-CBA-RG-mCherry/rAAV8-CBA- mRFP-IRES-TvA helpers and RABVΔG-eGFP tracers.

	Structure	AP* (mm)	ML* (mm)	DV* (mm)	*n*	Volume rAAV/RABV (μl)
Hippocampus	CA1/CA3	−2.18	± 2.1	1.9–1.5	3	1/1
	Dentate gyrus	−1.94	± 0.75	2.1–1.8	3	0.2/0.2
	Subiculum	−3.52	± 2.2	1.5–1.3	5	0.2/0.1
Rhinal Cortex	Lateral-, ento-, perirhinal	−4.84	−4.2	1.65–0.55	4	0.6/0.3
Retrosplenial cortex	Agranular and granular RSC	−1.46	± 0.25	0.25–0.1	3	0.5/0.5
Thalamus	Laterodorsal nuclei	−1.46	−1.5	2.25–1.9	4	0.2/0.2
	Ventrolateral nuclei	−1.58	−1	3.25–2.9	3	0.2/0.2

*AP, anterior-posterior; ML, mediolateral; DV, dorsoventral (all relative to Bregma).

### Histology

Mice were anesthetized with ketamine and xylazine (100 mg/kg and 10 mg/kg, respectively) and perfused transcardially with ice-cold 4% PFA (paraformaldehyde, Sigma-Aldrich) in PBS (pH 7.4). Brains were dissected and post-fixed for 1 h in 4% PFA in PBS, then cryoprotected in 30% sucrose in PBS at 4°C overnight. Brains were sliced in 35 μm thick sections using a Leica microtome. The sections were mounted with Roti-Mount FluorCare (Carl Roth) before analysis for fluorescence.

### Imaging

All images were acquired using a Leica TCS SP5 confocal laser scanning microscope (Leica DMI6000 B, Wetzlar, Germany) interfaced to a personal computer, running Leica Application Suite Advanced Fluorescence software (LAS AF 2.6). eGFP was excited with an Argon laser at 488 nm, while mCherry, tdTomato or mRFP were excited with a DPSS laser at 561 nm. Double-fluorescent images were obtained using the alternating acquisition mode. Sequential z-stacks were made for each section and crosstalk of the fluorophores was eliminated automatically with LAS AF software. Images were further analyzed using ImageJ. Schematic images show a quantitative localization from a combination of slices from ≥3 mice analyzed. Representative confocal images may be presented from different animals.

### Statistics

All statistical analyses were calculated with SigmaPlot software. Data were initially analyzed for normality by the Shapiro–Wilk test (*p* ≥ 0.05), then tested for equal variance with the Equal Variance Test (*p* ≥ 0.05). If data sets passed both tests, a *t*-test for comparison of tow groups or a One-Way analysis of variance (ANOVA; *post hoc* *t*-test) for comparison of more than two groups was used. If the normality tests failed, data sets were analyzed using the Mann-Whitney-*U* test for comparison of two groups. Significance for comparisons: *p ≤ 0.05, **p ≤ 0.01; ***p ≤ 0.001. Cell counts from specific areas of *n* ≥ 3 mice are presented as mean ± standard error of the mean (SEM) from all rabies injected mice. For counting, every second slice was imaged for eGFP^+^ cells followed by manual cell count using ImageJ.

## Results

### AAV-Mediated Polysynaptic Circuit Tracing of the Mouse Cerebellar-Hippocampal Connections

**Figure 1:**
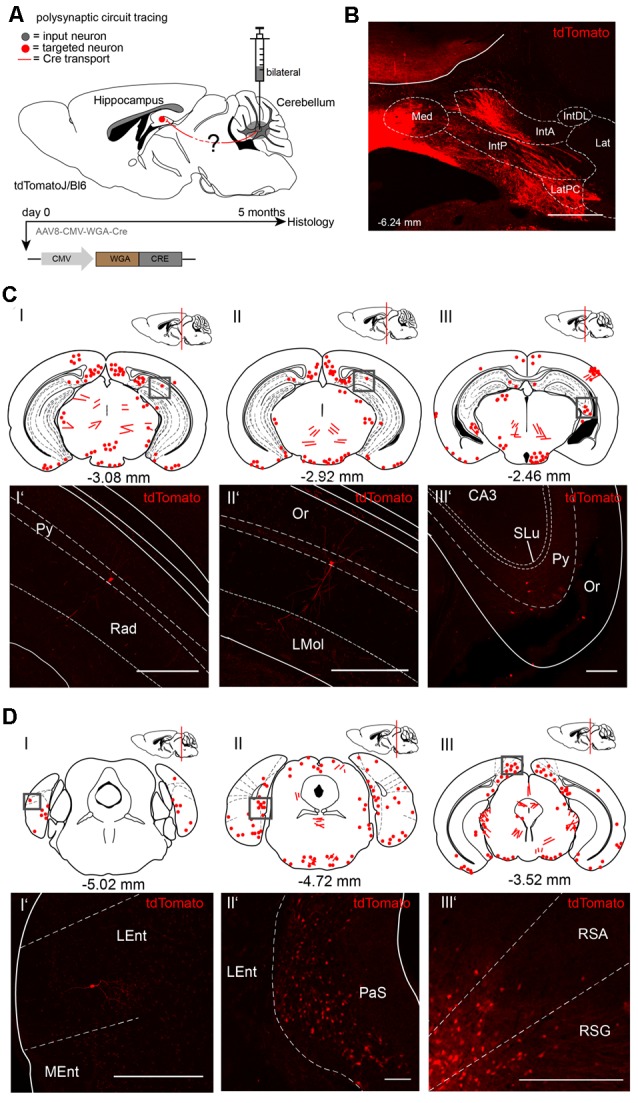
Mapping recombinant adeno-associated viruse (rAAV) mediated polysynaptic targets from the deep cerebellar nuclei (DCN) to the hippocampus using the tracer rAAV8-CMV-WGA-Cre in tdTomatoJ^+/+^ mice. **(A)** Schematic of sagittal section from an adult tdTomatoJ^+/+^ mouse brain (*n* = 4) injected bilaterally with 1 μl rAAV8-CMV-WGA-Cre in the DCN, (gray dot) for 5 months to trace the polysynaptic circuit (red line) to the hippocampus (red dot). **(B)** Confocal image depicting the injection site from an adult tdTomatoJ^+/+^ mouse brain in the DCN. Scale bar: 250 μm. **(CI–III)** Schematic of coronal sections showing quantitative localization of tdTomato^+^ neuron cell bodies (red dots) or neurites (red lines) at different distances from Bregma (−3.08 mm, −2.92 mm, −2.46 mm) after rAAV8-CMV-WAG-Cre injection in the DCN. Squared boxes represent confocal images presented in **(CI’–III’)**. **(CI’–III’)** Single tdTomato^+^ neurons were observed in the hippocampal pyramidal layer (Py, **CI’–III’**) or stratum oriens (Or, **CIII’**). Scale bars: 250 μm. **(D)** Schematic of coronal sections showing quantitative localization of tdTomato^+^ neuron cell bodies (red dots) or neurites (red lines) at different distances from Bregma (−5.02 mm, −4.72 mm, −3.52 mm) afterrAAV8-CMV-WAG-Cre injection in the DCN **(DI–III)**. Squared boxes represent confocal images presented in **(DI’–III’)**. **(DI’–III’)** tdTomato^+^ neurons were found in the parasubiculum (PaS, **DII’**) and in both retrosplenial granular (RSG, **DIII’**) and agranular (RSA, **DIII’**) cortex. Scale bars: 250 μm. IntA, anterior interposed cerebellar nucleus; IntP, posterior interposed cerebellar nucleus; Lat, lateral (dentate) cerebellar nucleus; LatPC, parvicellular Lat; Med, medial (fastigial) cerebellar nucleus. The mouse brains in this figure has been reproduced from Franklin and Paxinos (2001).

To visualize cerebellar-hippocampal connections, we first performed polysynaptic circuit tracing utilizing a Cre-recombinase encoding rAAV injected into the cerebellum or hippocampus of Cre-dependent tdTomato reporter mice (Madisen et al., 2010). In this virus, the transsynaptic transporter protein WGA was fused to the Cre recombinase (Figure 1A) and expressed under the control of the CMV promoter (Chamberlin et al., 1998). Since the expression efficiency of WGA-Cre may be region-specific, rAAV serotype 8 was used due to its higher efficacy to infect hippocampal and cerebellar neurons (Heinemann et al., 1991; Broekman et al., 2006). Initially, rAAV8-CMV-WGA-Cre was injected into the DCN bilaterally (1 μl, AP: −6.25 mm, MT: −0.7 mm, DV: 2.4–2.2 mm) from four tdTomato mice to induce tdTomato expression in WGA-Cre positive cells (Figure 1B). After 5 months of expression, single tdTomato^+^ neurons were detected in the lateral entorhinal cortex (Lent; Figures 1DI,I’), parasubiculum (PaS, Figures 1DII,II’), RSC (Figures 1DIII,III’) and also in parts of the hippocampus, including CA1 pyramidal neurons and in the stratum oriens layer (Figures 1CI’–III’) as represented by red dots and lines in the brain schemes. Fluorescent fibers and a few cells were detected in thalamic regions, including bilaterally in the medial geniculate nucleus (MGV, Figure 1DIII) and the magnocellular red nucleus (RMC; Supplementary Figure S1B). We also found fluorescent structures in the periaqueductal gray (DLPAG), the ventral secondary auditory cortex, frontal association cortex, olfactory bulb or the C3 posteromedial cortical amygdaloid nucleus (Supplementary Figure S1).

**Figure 2:**
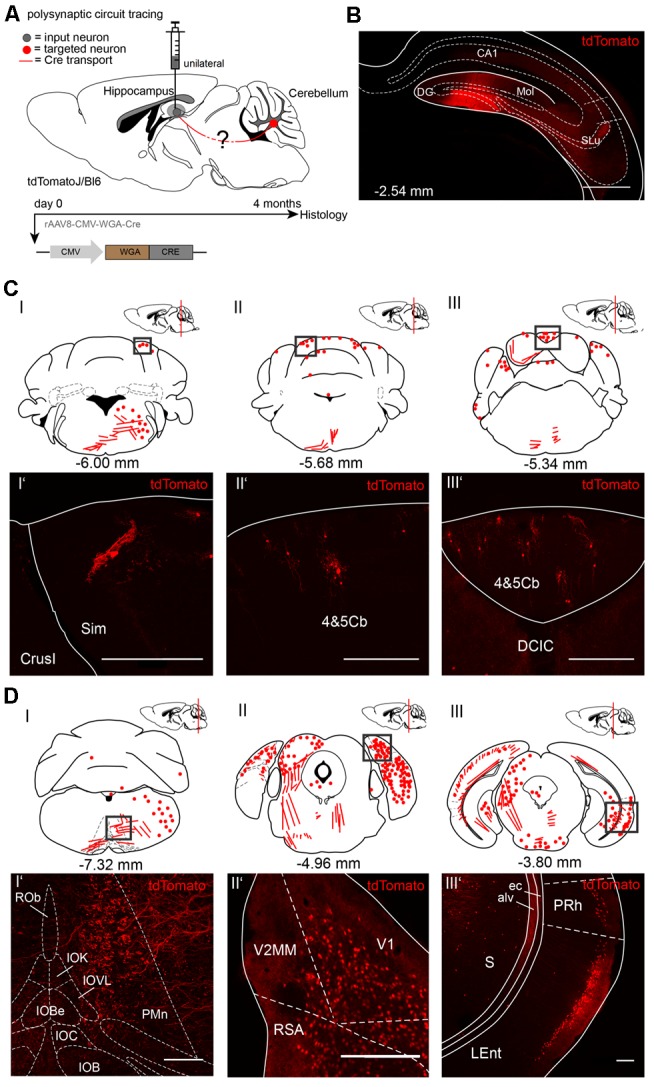
Mapping rAAV mediated polysynaptic targets from the dentate gyrus to cerebellum using the tracer rAAV8-CMV-WGA-Cre in tdTomatoJ^+/+^ mice. **(A)** Schematic of sagittal section from an adult tdTomatoJ^+/+^ mouse brain injected unilaterally with 1 μl rAAV8-CMV-WGA-Cre in the dentate gyrus (gray dot) for 4 months to trace the polysynaptic circuit (red line) to the cerebellum (red dot). **(B)** Confocal image of a coronal section from an adult tdTomatoJ^+/+^ mouse brain injected with rAAV8-CMV-WGA-Cre showing the injection site in the DG and hippocampus proper. Scale bar: 250 μm. **(CI–III)** Schematic of coronal sections showing quantitative localization of tdTomato^+^ neuron cell bodies (red dots) or neurites (red lines) at different distances from Bregma (−6.00 mm, −5.68 mm, −5.34 mm) after rAAV8-CMV-WGA-Cre injection in the dentate gyrus. Squared boxes represent confocal images presented in **(CI’–III’)**. **(CI’–III’)** Example single tdTomato^+^ neurons were imaged from the cerebellar lobulus simplex (Sim, **CI’**) and lobes 4 and 5, (4&5Cb, **CII’–III’**). Scale bars: 250 μm. **(DI–III)** Schematic of coronal sections showing quantitative localization of tdTomato^+^ neuron cell bodies (red dots) or neurites (red lines) at different distances from Bregma (−7.32 mm, −4.96 mm, −3.80 mm) after rAAV8-CMV-WGA-Cre injection in the dentate gyrus. Squared boxes represent confocal images presented in **(DI’–III’)**. **(DI’–III’)** Representative tdTomato^+^ axons were imaged from the ipsilateral side of injection from the paramedian reticular nucleus (PMn) and parts of the inferior olive (IO, **DI’**), while tdTomato^+^ cells were seen in primary visual cortex (V1) and retrosplenial agranular cortex (RSA, **DII’**), lateral entorhinal (LEnt) and perirhinal cortices (PRh, **DIII’**) and subiculum (S, **DIII’**). Scale bars: 150 μm.The mouse brains in this figure has been reproduced from Franklin and Paxinos (2001).

To trace from the hippocampus to the cerebellum we injected rAAV8-CMV-WGA-Cre unilaterally in the right dentate gyrus (DG, AP: −2.54 mm, ML: −1.5 mm, DV: 1.8 −1.6 mm, 1 μl) from three tdTomatoJ mice (Figure 2). After 4 months of expression tdTomato^+^ neurons in the cerebellum distributed equally over the ipsi-, and contralateral sides. We found cerebellar Purkinje cells classified by their typical morphology and location (Figure 2CI’). tdTomato^+^ neurons were additionally detected in the bilateral simple lobules and lobules 4 and 5 (Figures 2CII,II’,III,III’). Additionally, fluorescently labeled axons in the ipsilateral inferior olive (IO) and paramedian reticular nucleus (PMn; Figures 2CI,DI,I’), but not somata were observed. Numerous neurons were bilaterally expressing tdTomato in the visual and RSC, close to the cerebellum at −4.96 mm from Bregma (Figures 2DII–II’). However, the rhinal cortex was only stained on the ipsilateral and not the contralateral side (Figures 2DI,III–III’). In contrast, the contralateral central nucleus of the inferior colliculus (CIC), but not the ipsilateral side showed tdTomato^+^ neurons (Figure 2DII). Overall, we found differences in tdTomato expression in the thalamus following injection of WGA-Cre in the hippocampus vs. the cerebellar DCN. Injection in the cerebellar DCN showed more labeling in the thalamus, except for in the lateral posterior thalamus (Supplementary Figure S2C), suggesting the existence of different pathways connecting the cerebellum with the hippocampus in a loop, as was reported for motor-related areas (Dum and Strick, 2003; Kelly and Strick, 2003). We also found tdTomato+ neurites crossing the MGD/MGV (medial geniculate nucleus, dorsal and ventral parts; Supplementary Figure S2B) and dorsal raphe nucleus (DRN, Supplementary Figure S2B’). Some tdTomato+ expressing cells were seen in the medial septal nucleus (MS, Supplementary Figure S2D) and neurites in the lateral septal nucleus (LSI, Supplementary Figure S2D’).

**Figure 3:**
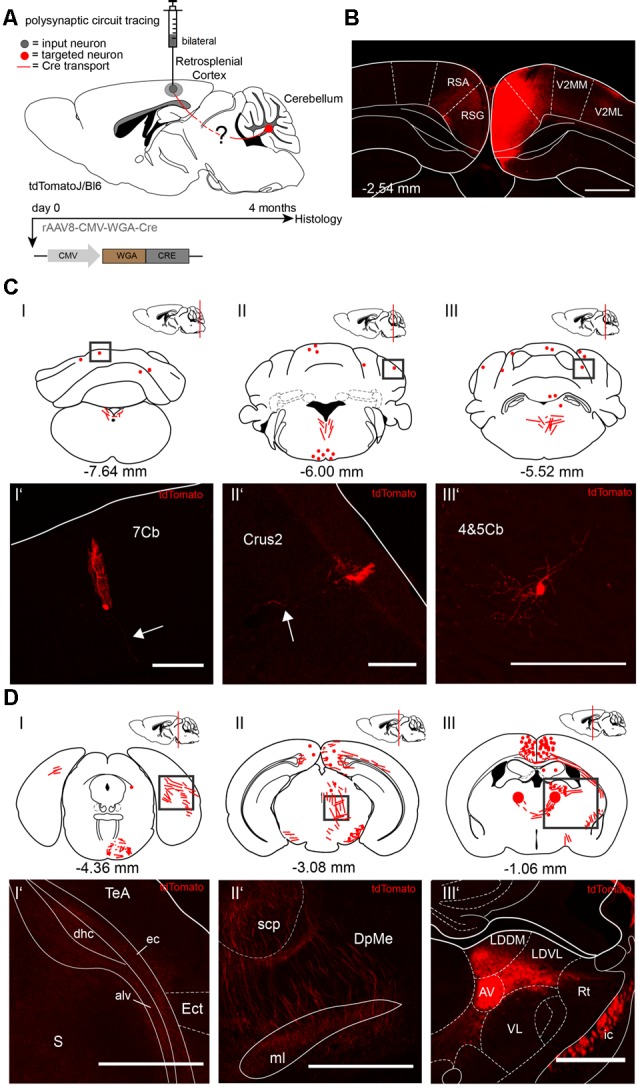
Mapping rAAV mediated polysynaptic targets from the retrosplenial cortex (RSC) to cerebellum using the tracer rAAV8-CMV-WGA-Cre in tdTomatoJ^+/+^ mice. **(A)** Schematic of sagittal section from an adult tdTomatoJ^+/+^ mouse brain injected bilaterally with each 1 μl rAAV8-CMV-WGA-Cre in the RSC, granular (RSG) and agranular (RSA) parts (gray dot) for 4 months to trace the polysynaptic circuit (red line) to the cerebellum (red dot). **(B)** Confocal image of a coronal section from an adult tdTomatoJ^+/+^ mouse brain injected with rAAV8-CMV-WGA-Cre showing the injection sites in the retrosplenial cortices (RSC). Scale bar: 250 μm. **(CI–III)** Schematic of coronal sections showing quantitative localization of tdTomato^+^ neuron cell bodies (red dots) or neurites (red lines) at different distances from Bregma (−7.64 mm, −6.00 mm, −5.52 mm) after rAAV8-CMV-WGA-Cre injection in the RSC. Squared boxes represent confocal images presented in **(CI’–III’)**. **(CI’–III’)** Example single tdTomato^+^ neurons were imaged from the cerebellar lobule 7 (7Cb, **CI’**), cerebellar Crus2 **(CII’)** and lobes 4 and 5, (4&5Cb, **CIII’**). Arrows pointing to tdTomato^+^ axons. Scale bars: 250 μm **(I’,II’)**, 100 μm (**I”’**). **(DI–III)** Schematic of coronal sections showing quantitative localization of tdTomato^+^ neuron cell bodies (red dots) or neurites (red lines) at different distances from Bregma (−4.36 mm, −3.08 mm, −1.06 mm) after rAAV8-CMV-WGA-Cre injection in the RSC. Squared boxes represent confocal images presented in **(DI’–III’)**. **(DI’–III’)** Example single tdTomato^+^ neurites were imaged from the left dorsal hippocampal commissure (dhc), alveus of the hippocampus (alv) and external capsule (ec, **DI’**), thalamic deep mesencephalic nucleus (DpME), superior cerebellar peduncle (scp) and medial lemniscus (ml, **DII’**), laterodorsal thalamic nuclei, dorsomedial (LDDM) and ventrolateral (LDVL), anteroventral thalamic nucleus (AV) and internal capsule (ic, **DIII’**) that are expressing tdTomato^+^ neurites. Scale bars: 150 μm. The mouse brains in this figure has been reproduced from Franklin and Paxinos (2001).

Since both injections of rAAV8-CMV-WGA-Cre virus in the cerebellar DCN (Figure 1) and hippocampus (Figure 2) demonstrated tdTomato^+^ neurons in the RSC, we injected the RSC, granular (RSG) and agranular (RSA) parts of two tdTomatoJ mice bilaterally (Figure 3) to further dissect the synaptic connections between the cerebellum and hippocampus (AP: −2.54 mm, ML: ± 0.3 mm, DV: 0.75–0.25 mm; 1 μl per site). Four months of expression and tracing time, we observed tdTomato^+^ cerebellar Purkinje cells (Figures 3CI’,II’) with their positive axons (arrows) and molecular layer interneurons (Figure 3CIII’) equally distributed over the cerebellar lobules (Figure 3C). We also detected tdTomato^+^ neurites in the right dorsal hippocampal commissure (dhc), alveus of the hippocampus (alv) and external capsule (ec) but not in the ectorhinal cortex (Ect) at −4.36 mm from Bregma, close to the cerebellum (Figures 3DI–I’). tdTomatoJ^+^ neurites crossed the superior cerebellar peduncle (scp), thalamic deep mesencephalic nucleus (DpMe) and medial lemniscus (ml) in the thalamus at −3.08 mm from Bregma (Figures 3DII–II’), while tdTomato^+^ cell somata were also seen in the right laterodorsal thalamic nuclei, dorsomedial (LDDM) and ventrolateral (LDVL) and bilateral anteroventral thalamic nuclei (AV; Figures 3DIII–III’).

**Figure 4:**
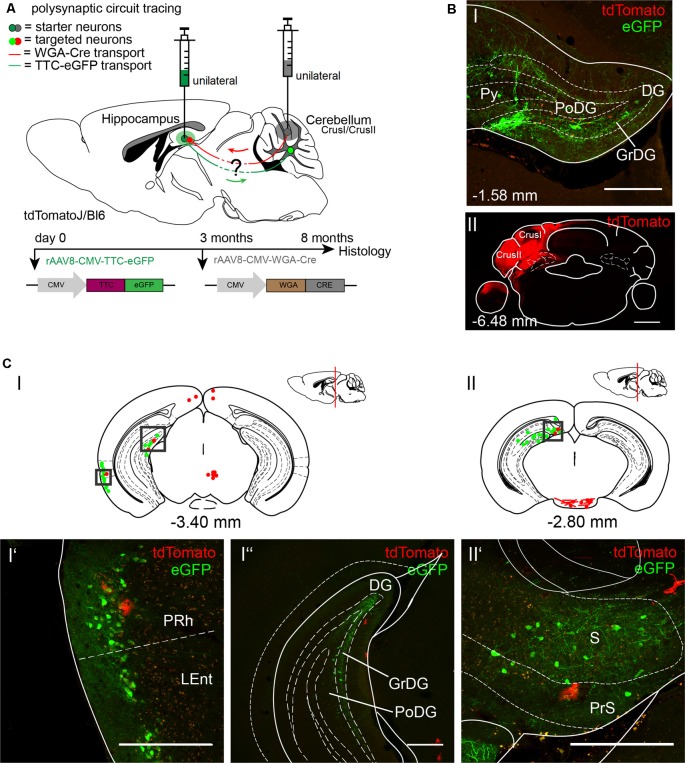
Mapping the shared circuit between the dentate gyrus and cerebellar cortex with an rAAV retrograde and anterograde tracer. **(A)** Schematic of sagittal section from an adult tdTomatoJ^+/+^ mouse brain injected with 1 μl of the retrograde specific rAAV8-CMV-TTC-eGFP (green dot) in the dentate gyrus for 8 months and rAAV8-CMV-WGA-Cre (1 μl) in the cerebellar CrusI/CrusII (gray dot) for 5 months to determine the common polysynaptic circuits (red and green lines). The viruses were injected at different time points to allow better expression of rAAV8-CMV-TTC-eGFP. **(B)** Confocal image of a coronal section from an adult tdTomatoJ^+/+^ mouse brain injected unilaterally with rAAV8-CMV-TTC-eGFP in the dentate gyrus [DG, green, upper **(I)**] and rAAV8-CMV-WGA-Cre in cerebellar CrusI/CrusII [red, lower **(II)**]. Cell bodies stained with tdTomato are evident in the dentate gyrus. Scale bars: 250 μm. **(CI,II)** Schematic of coronal sections showing quantitative localization of tdTomato^+^ (red dots) and eGFP^+^ (green dots) neuronal cell bodies or neurites (red lines) at different distances from Bregma (−3.40 mm, −2.80 mm). Squared boxes represent images in **(CI’–CII’)**. **(CI’–II’)** Example images from squared boxes in **(CI’–II’)** of tdTomato^+^ and eGFP^+^ positive cell bodies in perirhinal cortex (PRh, **I’**), dentate gyrus (DG, **I”**) and presubiculum (PrS, **II’**) where circuits are shared. Scale bars: 250 μm. The mouse brains in this figure has been reproduced from Franklin and Paxinos (2001).

To further explore the cerebellar-hippocampal circuitry as reported previously for motor-related areas, we injected the anterograde tracer rAAV8-CMV-WGA-Cre in left cerebellar Crus1/Crus2 (Figures 4A,BII; AP: −6.4 mm, ML: −2.7 mm, DV: 1.7–1.07), which may be involved in sequence-based navigation, and the retrograde tracer rAAV8-CMV-TTC-eGFP in the left DG of tdTomatoJ mice (Figures 4A,BI; Burguière et al., 2010; Iglói et al., 2015). Since rAAV8-CMV-TTC-eGFP requires a longer expression time in the hippocampus compared to WGA-Cre, it was initially injected 3 months prior to rAAV8-CMV-WGA-Cre (Figure 4A). Unilateral injection of rAAV8-CMV-TTC-eGFP in the DG resulted in eGFP^+^ neurons in the DG (Figure 4CI”), perirhinal (PRh) and LEnt (Figures 4CI,CI’), the subiculum (S) and presubiculum (PrS; Figures 4CII,CII’), but failed to label structures beyond these areas. The injection of WGA-Cre in cerebellar Crus1/Crus2 resulted in tdTomato^+^ cells, likely astrocytes, in similar areas such as the PRh (Figure 4CI’) and PrS (Figure 4CII’), but no neurons were identified expressing both fluorescent proteins.

### Retrograde Monosynaptic Tracing of the Mouse Hippocampal-Thalamic-Cerebellar Circuitry Using Deletion Mutant Rabies Virus

**Figure 5:**
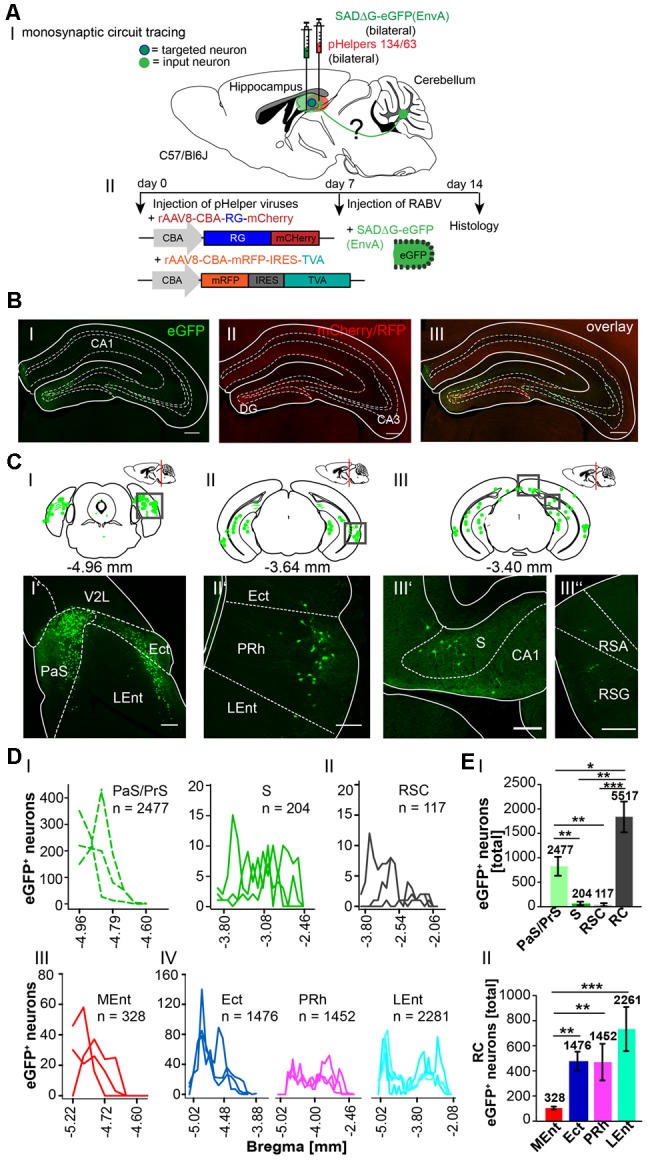
Inputs into the hippocampus using a monosynaptic, retrograde modified RABV tracer. **(AI)** Schematic of sagittal section from an adult mouse brain injected with the retrograde specific modified RABV tracer (green dots) and pH in the hippocampus (1 μl). The pHelper viruses rAAV8-CBA-RG-mCherry and rAAV8-CBA-mRFP-IRES-TVA were injected 7 days prior to the SADΔG-eGFP (EnvA) rabies virus (1:2 ratio, 1 μl/site). Animals were sacrificed 7 days after RABV injection. **(AII)** Overview of the bilaterally injected hippocampus. The framed area represents the hippocampus shown in **(B)**. Scale bar: 1 mm. **(B)** Higher magnification of the right injection site (Bregma −1.94 mm) showing hippocampal neurons infected with the Helper virus (RFP^+^; **BI**) or rabies virus (eGFP^+^; **BII**). Scale bar: 250 μm. Merge **(BIII)** of images in **(B’)** and **(B”)** showing double-fluorescent neurons in the dentate gyrus. Scale bar: 250 μm. **(CI–III)** Schematic of coronal sections showing quantitative localization of eGFP^+^ (green dots) neuronal cell bodies at different distances from Bregma (−4.96 mm, −3.64 mm, −3.40 mm) in all analyzed mice (*n* = 3). Squared boxes represent images in **(CI’–III”)**. **(CI’–III”)** Example images from squared boxes in **(CI–III)** demonstrate that eGFP^+^ cell bodies represent inputs into the dentate gyrus from the parasubiculum (PaS, **I’**), ectorhinal cortex (Ect, **I’**), lateral entorhinal cortex (LEnt, **I’**), perirhinal cortex (PRh, **II’**), subiculum (S, **III’**) and RSC, agranular and granular parts (RSA, RSG, **III”**). **(D)** Graphical illustration showing the representative number of eGFP^+^ neurons found at different mm from Bregma in Para-, and Pre-subiculum, S (PaS, Pre; **DI**) and the RSC **(DII)**, with each line representing the count in one mouse. **(DI,II)** A total of 2,477 eGFP expressing cells were count in PaS/Pre of *n* = 3 mice (dotted lines), while only 204 eGFP^+^ cells were detected in the S **(DI)** and 117 cells in the RSC **(DII)**.A total of 2281 eGFP+ cells were found in the Ent, followed by 1476 eGFP+ cells in the Ect and 1452 cells in the PRh (DIV).**(EI)** Statistical analysis comparing all input areas to the Hippocampus. With a total of 5,517 eGFP^+^ cells, the rhinal cortex (RC) provides significantly more input to the murine Hippocampus compared to RSC (*p* = 0.001, *t*-test), S (*p* = 0.002, *t*-test) and Pre/PaS (*p* = 0.016, *t*-test). Pre/PaS significantly increased projections compared to S (*p* = 0.003, *t*-test) and RSC (*p* = 0.002, *t*-test). **(DIII)** Graphical illustration showing the representative number of eGFP^+^ neurons found at different mm from Bregma in Ect (1,476 neurons), PRh (1,452), LEnt (2,261) and medial entorhinal cortex (MEnt, 328).All areas that provide monosynaptic input to the hippocampus as revealed by eGFP+ cells. The RC provides strongest synaptic input to the hippocampus compared to RSC (*p* = 0.001, *t*-test), S (*p* = 0.002, *t*-test) and PaS/PrS (*p* = 0.016, *t*-test). A total of 2477 eGFP+ cells were found in the PaS/PrS, which is significantly more compared to S (*p* = 0.003, *t*-test) and RSC (*p* = 0.002, *t*-test).**(EII)** Total number of eGFP^+^ neurons in the rhinal cortex of *n* = 3 mice showing significantly more projections (One-way analysis of variance, ANOVA), *post hoc* Tukey (*p* < 0.05) from the Ect (1,476 cells) PRh (1,422) and LEnt (2,261) compared to the MEnt (328). Significance for comparisons: *p ≤ 0.05; **p ≤ 0.01; ***p ≤ 0.001.DG, dentate gyrus; Ect, ectorhinal cortex; LEnt, lateral entorhinal cortex; MEnt, medial entorhinal cortex; PaS, parasubiculum; PRh, perirhinal cortex; PrS, presubiculum; Py, pyramidal cell layer of the hippocampus; RSA, agranular retrosplenial cortex; RSC, retrosplenial cortex; RSG, granular retrosplenial cortex; RC, rhinal cortex; S, subiculum; V2L, secondary visual cortex, lateral area. The mouse brains in this figure has been reproduced from Franklin and Paxinos (2001).

To determine the sequence of connections between the cerebellum and hippocampus in more detail, we used the retrograde mono-transsynaptic tracer RABVΔG-eGFP (EnvA), allowing a retrograde step-by-step tracings to the cerebellum, starting from the hippocampus (Wickersham et al., 2007a,b). To allow efficient infection, as well as transsynaptic traversal of neurons by our modified RABV we first infected the hippocampus proper and DG of 6 C57/Bl6 mice (three mice/area) with two rAAV expressing the rabies virus glycoprotein and the TVA receptor (Niedworok et al., 2012). One week later we injected into the same site RABVΔG-EGFP (EnvA). We injected both the hippocampus proper and DG for two reasons (Figure 5). First, the connectivity of this structure is well described and serves as a valuable control for obtained tracings results. Second, we wanted to test a possible existence of a weak but monosynaptic cerebellar projection to the hippocampus. After 1 week of RABV expression in the hippocampus (DG and CA1/CA3, see Table 2 in the “Materials and Methods” section for coordinates), eGFP^+^ neurons appeared in the rhinal cortex (RC), including entorhinal (Ent), PRh, LEnt and parts of the medial entorhinal (MEnt) cortex, PrS and S (Figure 5C), lateral and medial supramammillary nuclei, mammillary tract and medial septal nucleus, as well as nuclei in the dorsal raphe and horizontal limb of the diagonal band (HDB; Supplementary Figure S3). We found significantly more eGFP^+^ neurons in the RC (5,517 cells) compared to RSC, S and PrS/PaS (*p* = 0.001; *p* = 0.002; *p* = 0.016, each *t*-test *n* = 3; Figures 5DI,EI). Graphical illustration of the distribution of eGFP^+^ neurons depending to their distance to Bregma of the RC showed that the LEnt provides strongest synaptic input to the hippocampus (2,261 cells), followed by Ect (1,476), PRh (1,452) and MEnt (328; Figures 5DIII,DIV; analyzed with One-way ANOVA). Notably, we did not detect fluorescence in the cerebellum or in the thalamus.

**Figure 6:**
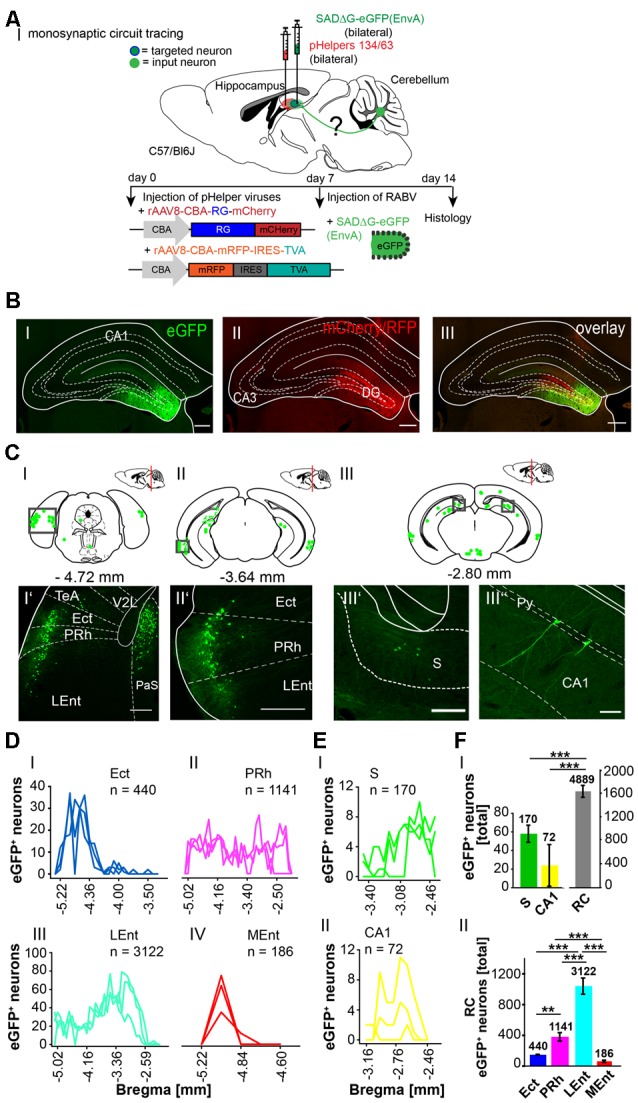
Mapping monosynaptic input areas to the dentate gyrus in C57/Bl6 mice using the retrograde SADΔG-eGFP tracer. **(AI)** Scheme of a sagittal section from an adult mouse brain injected with the retrograde specific RABV tracer (green circle) in the dentate gyrus of C57/Bl6 mice (*n* = 3). The pHelper viruses rAAV8-CBA-RG-mCherry and rAAV8-CBA-mRFP-IRES-TVA (1:2 ratio, 0.2 μl) were injected 7 days prior to the SADΔG-eGFP (EnvA) rabies virus (0.2 μl). **(B)** Confocal image showing one exemplary injection site in the left DG with rabies virus (eGFP, **BI**) and pHelpers (mcherry, **BII**) in the DG. Overlay reveals coinfected starter neurons for monosynaptic retrograde tracing **(BIII)**. Scale bars: 250 μm. **(CI–III)** Coronal brain sections at −4.72 mm, −3.64 mm and −2.80 mm from Bregma depicting eGFP^+^ neurons (green dots) by retrograde monosynaptic transport from the DG. **(CI–III”)** Confocal image from boxed area presented in CI representing several eGFP^+^ neurons in the outer layers of the RC, including Ect, PRh and LEnt **(CI’–II’)**, the S **(CIII’)** and pyramidal CA1 cells of the hippocampus **(CIII”)**, representing input to the DG. Scale bars: 250 μm. **(D)** Line plots mapping the individual distribution of eGFP^+^ neurons by retrograde monosynaptic transport from the DG to the Ect **(DI)**, PRh **(DII)**, LEnt **(DIII)** and MEnt **(DIV)** in all mice. Each line represents the count of eGFP^+^ cell bodies in one mouse. **(E)** Line plots mapping the individual distribution of eGFP^+^ neurons by retrograde monosynaptic transport from the DG to the S **(EI)** and CA1 **(EII)**. **(FI)** Compared to S and CA1, the RC provides the most input to the DG revealed by monosynaptic retrograde transport (*t*-test, for S: *p* ≤ 0.001; for CA1 *p* ≤ 0.001). **(FII)** Within the RC, the LEnt forms significantly more synapses with the DG with a total of 3,122 eGFP^+^ cells compared to PRh (*p* ≤ 0.001), Ect (*p* ≤ 0.001) and MEnt (*p* ≤ 0.001, all One-Way-ANOVA). In the PRh a total of 1,141 cells was counted and is the second strongest input source to the DG compared to Ect (*p* = 0.006) and MEnt (*p* = 0.001, One-Way-ANOVA).Significance for comparisons: **p ≤ 0.01; ***p ≤ 0.001.DG, dentate gyrus; Ect, ectorhinal cortex; LEnt, lateral entorhinal cortex; MEnt, medial entorhinal cortex; PRh, perirhinal cortex; Py, pyramidal cell layer of the hippocampus; RC, rhinal cortex; S, subiculum; TeA, temporal association cortex; V2L, secondary visual cortex, lateral area. The mouse brains in this figure has been reproduced from Franklin and Paxinos (2001).

Since the hippocampus receives inputs predominantly *via* the dentate gyrus, we next bilaterally injected only the DG with the above-mentioned viruses (each 0.2 μl) to differentiate the synaptic inputs to the hippocampus proper and DG (Figure 6). Similarly, eGFP^+^ neurons were found in the same areas as after rAAV8-CMV-WGA-Cre injections into the CA1/CA3 region, including PRh and LEnt (Figures 6CI’,II’) but also ectorhinal cortex (Repapi et al., 2009), PaS (Figure 6CI’), S and CA1 (Figures 6CIII’,III”), suggesting a monosynaptic input from these regions to the DG. We also found eGFP^+^ cells in the lateral and medial supramammillary nuclei medial raphe nucleus and nuclei in the horizontal limb of the diagonal band (Supplementary Figure S4). We confirmed that neurons of the PRh synapse onto DG and the CA1 neurons, as previously reported by Agster and Burwell (2013). Projections from Ect, LEnt and MEnt to the DG have already been described in mice, as well as the input from the supramammillary nuclei but a monosynaptic projection from the PaS to the DG as observed here has not been reported (Figure 6CI’; Vertes and McKenna, 2000; van Groen et al., 2002b,c; Hartley et al., 2013). Thus, injections of modified RABV in the hippocampus and DG both confirmed already known projections, but also revealed a new projection from the PaS to the DG. Analysis revealed that the DG receives most input from the RC (4,889 cells in ≥3 mice, Figure 6F), when compared to CA1 and S (*p* < 0.001, *t*-test), which is in accordance with our results presented in Figure 5D. With these findings, we show that RC input to the hippocampus is delivered *via* the DG. Within the RC, the LEnt contributed the most synaptic input to the DG (*n* = 3,122 cells) compared to the PRh (*n* = 1,141 cells), Ect (*n* = 440 cells) and MEnt (*n* = 186 cells, One-Way-ANOVA, Figures 6D,FII).

**Figure 7:**
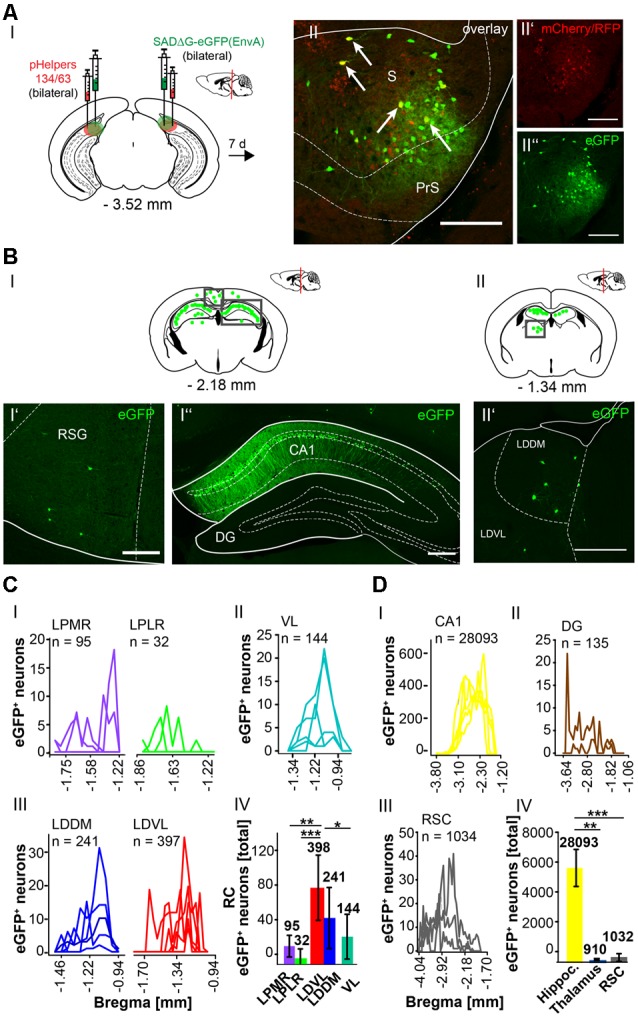
Identifying a monosynaptic circuit to the subiculum from the thalamus using the retrograde, modified RABV tracer. **(A)** Schematic of a coronal section at Bregma −3.52 mm **(AI)** from an adult mouse brain injected bilaterally with the retrograde specific, modified RABV tracer (green circle, 0.1 μl, **AII”**) and pHelper mixture (red circle, **AII’**) in the subiculum (S) and presubiculum (PrS) of *n* = 5 mice. The pHelper viruses rAAV8-CBA-RG-mCherry and rAAV8-CBA-mRFP-IRES-TVA (1:2 ratio, 0.2 μl) were injected 7 days prior to the SADΔG-eGFP (EnvA) rabies virus. Animals were sacrificed 7 days after modified RABV injection. **(AII)** Confocal image showing co-expression of both pHelper and rabies viruses. Scale bars: 250 μm. **(BI)** Coronal brain scheme at −2.18 mm from Bregma showing eGFP^+^ neurons (green dots) by monosynaptic retrograde transport from the S and PrS to the (RSG, **BI’**) and hippocampal CA1 **(BI”)**. **(BII)** Coronal brain section at Bregma −1.34 mm depicting eGFP^+^ neurons by monosynaptic retrograde transport from the S and PrS to hippocampal CA1 and laterodorsal and ventrolateral thalamus (LDDM/LDVL, **BII’**). Scale bar: 250 μm. **(CI–III)** Line plots mapping the individual distribution of eGFP^+^ neurons by retrograde monosynaptic transport from the S and PrS to the lateral posterior thalamic nucleus, mediorostral and laterorostral parts (LPMR/LPLR, **CI**), ventrolateral thalamus (VL, **CII**) and LDDM and LDVL **(CIII). (CIV)** With total 398 eGFP^+^ cells, the LDVL provides strongest synaptic input to the S and PrS compared to LPMR (*p* ≤ 0.001), LPLR (*p* = 0.006) and VL (*p* = 0.024, analyzed with One-Way-ANOVA; **DI**) Line plots mapping the individual distribution of eGFP^+^ neurons by retrograde monosynaptic transport from the S and PrS to the CA1 **(DI)** and DG **(DII)** region of the hippocampus and to the RSC **(DIII)**. eGFP^+^ cells in the DG were found in two of the five analyzed mice. **(DIV)** Compared to the thalamic nuclei **(CI–III)** and RSC **(DIII)**, the hippocampus exhibited a total of 28,228 eGFP^+^ cells, which is significantly more than in the thalamus (*p* = 0.008, Mann-Whitney *U*) and RSC (*p* ≤ 0.001, *t*-test).Significance for comparisons: *p ≤ 0.05; **p ≤ 0.01; ***p ≤ 0.001.DG, dentate gyrus; LDDM, dorsomedial laterodorsal thalamic nucleus; LDVL, ventrolateral laterodorsal thalamic nucleus; LPLR, laterorostral lateral posterior thalamic nucleus; LPMR, mediorostral lateral posterior thalamic nucleus; PrS, Presubiculum; RSC, retrosplenial cortex; RSG, granular retrosplenial cortex; S, Subiculum; VL, ventrolateral thalamus. The mouse brains in this figure has been reproduced from Franklin and Paxinos (2001).

**Figure 8:**
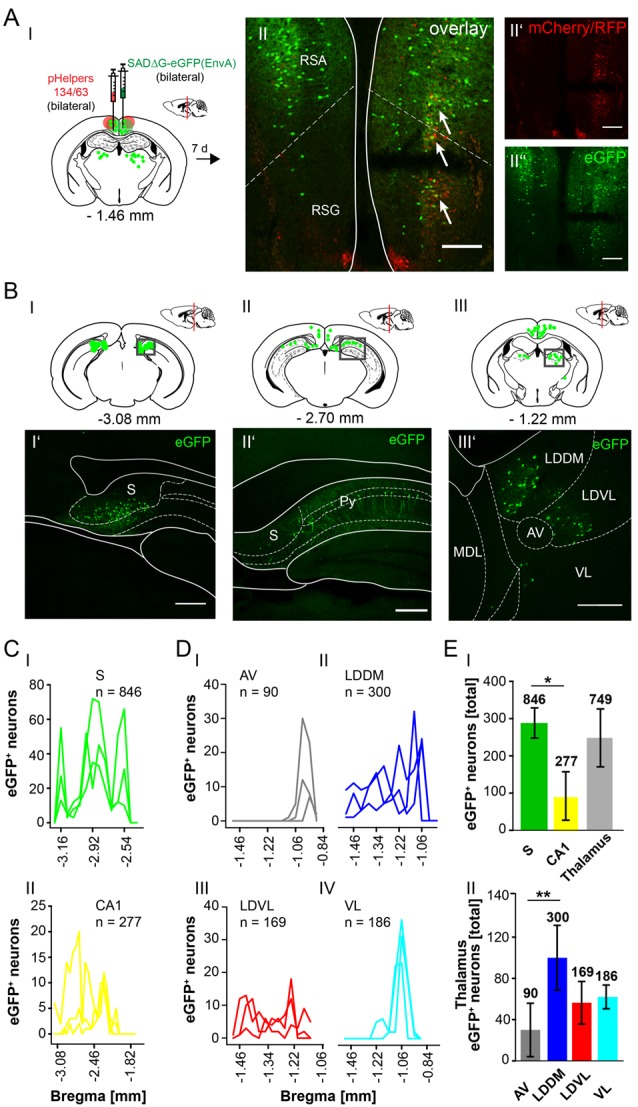
Mapping the circuit between the RSC and thalamus. **(AI)** Schematic of coronal section at Bregma −1.46 mm and confocal images of rabies infection site **(AII”)** and pHelpers **(AII’)** depicting virus spread to the RSA and RSG areas **(AII)**. Green dots represent the retrograde specific, modified RABV tracer expression. The pHelper viruses rAAV8-CBA-RG-mCherry and rAAV8-CBA-mRFP-IRES-TVA (1:2 ratio, 0.5 μl) were injected 7 days prior to the SADΔG-eGFP (EnvA) rabies virus (0.5 μl). Mice (*n* = 3) were sacrificed 7 days after modified RABV injection. Double infected neurons represent starter neurons for retrograde transport in RSA and RSG (arrows, **AII**). Scale bars: 150 μm**. (BI–III)** Coronal brain sections at Bregma −3.08 mm **(BI)**, −2.70 mm **(BII)** and −1.22 mm **(BIII)** depicting eGFP^+^ neurons (green dots) undergoing monosynaptic retrograde transport from the RSC. **(BI’–III’)** Images from boxed areas in **(BI–BIII)**. eGFP^+^ neurons outside the injection site were detected in the S **(BI’)**, CA1 pyramidal cells **(BII’)** and in the LDDM and LDVL **(BIII’)**. Scale bars: 250 μm. **(C)** Line plots mapping the distribution of eGFP^+^ neurons per mouse by retrograde monosynaptic transport from the RSC to the S **(CI)** and CA1 **(CII)** and their distance to Bregma. **(D)** Line plots mapping the distribution of eGFP^+^ neurons per mouse by retrograde monosynaptic transport from the RSC to AV **(DI)**, LDDM **(DII)**, LDVL **(DIII)** and VL **(DIV)**. **(EI)** A total of 846 eGFP^+^ cells was found in all mice in the S, showing significantly more input from the S to the RSC compared to the CA1 region (*p* = 0.011, *t*-test), but not the thalamus (*p* = 0.473, *t*-test). **(EII)** Within the thalamus, the LDDM showed significantly more eGFP^+^ cells then the AV (*p* = 0.027, One-Way-ANOVA), but no other significance was found between the nuclei (LDVL *p* = 0.185; VL *p* = 0.273). Significance for comparisons: *p ≤ 0.05; **p ≤ 0.01. AV, anteroventral thalamic nucleus; DLG, dorsal lateral geniculate nucleus; LDDM, dorsomedial laterodorsal thalamic nucleus; LDVL, ventrolateral laterodorsal thalamic nucleus; LPLR, laterorostral lateral posterior thalamic nucleus; LPMR, mediorostral lateral posterior thalamic nucleus; MDL, lateral mediodorsal thalamic nucleus; Po, posterior thalamic nuclear group; Py, pyramidal cell layer of the hippocampus; RSA, agranular retrosplenial cortex; RSG, granular retrosplenial cortex; S, Subiculum; VL, ventrolateral thalamic nucleus. The mouse brains in this figure has been reproduced from Franklin and Paxinos (2001).

**Figure 9:**
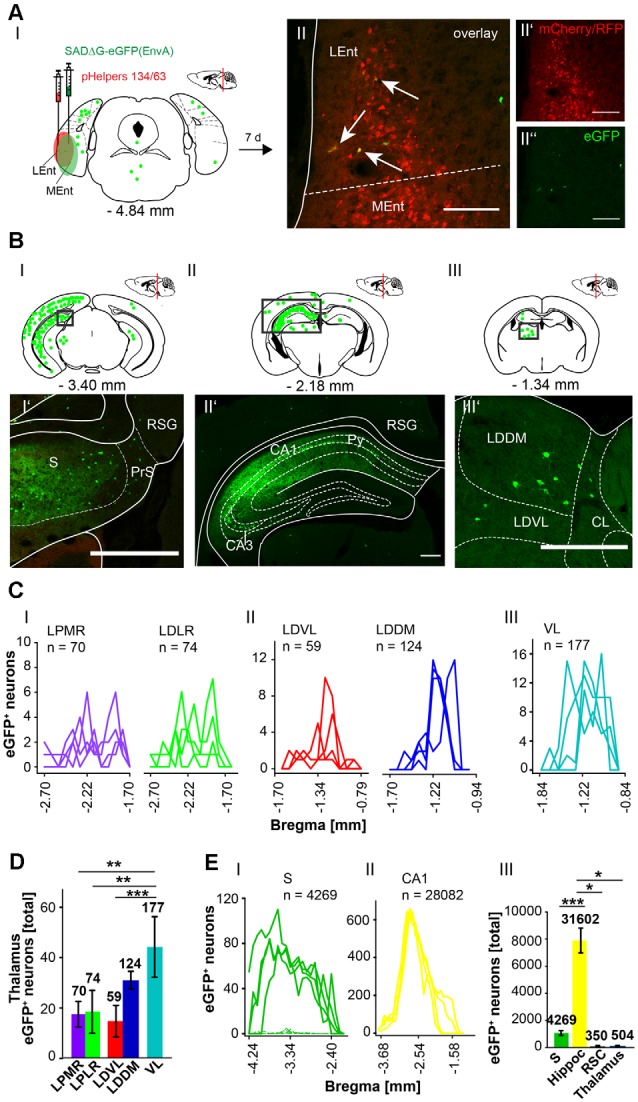
Identifying inputs into the rhinal cortex from the subiculum, hippocampal CA1 region and thalamus using the retrograde, modified RABV tracer. **(AI)** Schematic of coronal section at Bregma −4.84 mm depicting the injection site in *n* = 4 mice. Confocal images show exemplary infection of pHelpers **(AII’)** and rabies virus **(AII”)** depicting virus spread in the rhinal cortex including lateral (Lent) and medial (MEnt) entorhinal cortices (RC). Overlay reveals coinfected starter cells (arrows in **AII**) of retrograde monosynaptic transport. Green dots represent the retrograde specific, modified RABV tracer expression (0.3 μl). The pHelper viruses rAAV8-CBA-RG-mCherry and rAAV8-CBA-mRFP-IRES-TVA (1:2 ratio, 0.6 μl) were injected 7 days prior to the SADΔG-eGFP (EnvA) rabies virus. Mice were sacrificed 7 days after RABV injection. Scale bar: 150 μm. **(BI–III)** Coronal brain section at Bregma −3.40 mm **(BI)** and −2.18 mm **(BII)** and **(BIII)** −1.34 mm depicting eGFP^+^ neurons (green dots) by monosynaptic retrograde transport from the rhinal cortex. Rabies infected cells were present in the S **(BI’)**, CA1 region of ipsilateral hippocampus and RSC **(BII’)**. **(BIII’)** Single eGFP^+^ neurons were found in ipsilateral LDDM and LDVL, but also centrolateral thalamic nucleus (CL) Scale bars: 250 μm. **(C)** Line plots mapping the distribution of eGFP^+^ neurons per mouse by retrograde monosynaptic transport from the RC to different thalamic nuclei. eGFP^+^ cells were found in the mediorostral and laterorostral lateral posterior (LPMR/LPLR, **CI**), ventrolateral and dorsomedial laterodorsal (LDVL/LDDM, **CII**) and ventrolateral (VL, **CIII**) thalamic nuclei. **(D)** Compared to all thalamic nuclei, the VL had a total of 177 eGFP^+^ cells and thus provides strongest monosynaptic input to the RC compared to LDVL (*p* ≤ 0.001), LPLR (*p* = 0.002) and LPMR (*p* = 0.002; One-Way-ANOVA). **(E)** Line plots mapping the distribution of eGFP^+^ neurons per mouse by retrograde monosynaptic transport from the RC to S **(EI)** and CA1 **(EII)**. **(EIII)** Compared to all areas where eGFP^+^ cells were found, the hippocampus (including CA1, DG and CA3 (shown in Supplementary Figures S6, S7) provides strong synaptic input to the RC revealed by monosynaptic retrograde transport compared to S (*p* ≤ 0.001, *t*-test), RSC (*p* = 0.029, Mann-Whitney-*U*-test) and thalamus (*p* = 0.029, Mann-Whitney-*U*-test).Significance for comparisons: *p ≤ 0.05; **p ≤ 0.01; ***p ≤ 0.001.CL, centrolateral thalamic nucleus; LDDM, dorsomedial laterodorsal thalamic nucleus; LEnt, lateral entorhinal cortex; LDVL, ventrolateral laterodorsal thalamic nucleus; LPLR, laterorostral lateral posterior thalamic nucleus; LPMR, mediorostral lateral posterior thalamic nucleus; MEnt, mendial entorhinal cortex; PrS, Presubiculum; Py, pyramidal cell layer of the hippocampus; RSC, Retrosplenial cortex; RSG, granular retrosplenial cortex; S, Subiculum; VL, ventrolateral thalamic nucleus. The mouse brains in this figure has been reproduced from Franklin and Paxinos (2001).

To further explore whether the cerebellum synapses directly on other hippocampal input regions including the S (Figure 7), RSC (Figure 8) and Ent (Figure 9), the modified RABV was injected in these areas of C57/Bl6 mice. Bilateral injection of RABVΔG-eGFP virus in the S (AP: −3.52 mm, ML: ± 2.2 mm; DV: 1.5–1.3 mm, *n* = 5) resulted in double-fluorescent neurons in the subiculum resulting from infection from both rAAV helper viruses (mCherry/RFP, 0.2 μl) and rabies virus (eGFP, 0.1 μl; Figure 7AII). The laterodorsal thalamic nucleus, dorsomedial (LDDM) and ventrolateral parts (LDVL) showed eGFP^+^ neurons, representing direct monosynaptic input to the S (Figure 7BII’). Only 2–5 eGFP^+^ cells were observed in the PRh of each S injected mouse, thus confirming sparse input from the RC to the S. Additionally, we found a total of 28,093 CA1 pyramidal cells at various distances from Bregma (Figures 7BI”,DI) and 1,034 cells in the RSC (Figures 7BI’,DIII). To our knowledge, the LDDM has not been reported before to synapse on the subiculum directly and a total of 639 cells were counted in all mice in the laterodorsal thalamus. These are significantly more compared to the LPMR (*p* ≤ 0.001) and LPLR (*p* = 0.006) or VL (*p* = 0.024, One-Way-ANOVA, Figure 7CIV).

The RSC has been previously proposed to connect the cerebellum with the hippocampus *via* a polysynaptic circuitry (Rochefort et al., 2013). To identify these circuits, we bilaterally injected the monosynaptic RABVΔG-eGFP (0.5 μl each rAAV helpers and RABV) in the retrosplenial cortex, granular (RSG) and agranular (RSA) areas (Figure 8A) at −1.46 mm from Bregma (ML: ± 0.25 mm; DV: 0.25–0.1 mm; *n* = 3). An average of nine neurons in both regions were co-expressing both helper (mCh/RFP) and RABV (eGFP, Figure 8AII). Although there was no tracer observed in the cerebellum, staining in the left LDDM and LDVL, as well as in the ventrolateral thalamic nucleus (VL) at −1.22 mm from Bregma (Figure 8BIII’) was evident, indicating a direct monosynaptic input from the thalamus to the RSA as reported for the LDDM in rats (Sripanidkulchai and Wyss, 1986). In all mice analyzed, the thalamic nuclei equally project to the RSC, except the AV, which has significantly less eGFP^+^ cells than the LDDM (*p* = 0.027, One-Way-ANOVA, Figure 8EII). We also found eGFP^+^ cells in the S and hippocampal CA1 (Figures 8BI’,II’), with the S forming more synapses on the RSC than the CA1 cells (*p* = 0.011, *t*-test). Additionally, few eGFP^+^ neurons in the medial and median raphe nuclei, lateral supramammillary nucleus, dorsal secondary auditory cortex and the pontine reticular nucleus (Supplementary Figure S5) were detected.

Since the RC provides strong monosynaptic input to the dentate gyrus in mice (Hartley et al., 2013), we wanted to test the possibility of a monosynaptic connection between the RC and the cerebellum. Injection of the modified RABV (0.3 μl) in the left rhinal cortex at −4.84 mm from Bregma (ML: −4.2 mm, DV: 1.65–0.55, *n* = 4) revealed co-expressing neurons distributed in the LEnt (Figure 9AII). Many eGFP+ neurons were observed at the ipsilateral side, predominantly in hippocampal CA1, CA2 and CA3 region (Figures 9BI,II,II’) and S (Figure 9BI’) and RSC (Figure 9BII), indicating direct monosynaptic input from these areas to the RC. eGFP^+^ expressing neurons were also detected in the DG bilaterally and in the ipsilateral LPMR and medial geniculate nucleus, dorsal (MGD) and ventral parts (MGV; Supplementary Figure S6). Furthermore, the RC receives less synaptic input from the Barrington’s nucleus and the gigantocellular reticular nucleus in the pons, the ipsilateral motor cortex M2, the medial septal nucleus, the lambdoid zone and the ipsilateral nucleus of the horizontal limb of the diagonal band (Supplementary Figure S6). eGFP^+^ cells were also detected and analyzed in the thalamus, including LDDM/LDVL and VL (Figures 9BIII’,CIII). Within the thalamus, the VL forms more synapses with the RC compared to LDVL (*p* ≤ 0.001), LPMR (*p* = 0.002) and LPLR (*p* = 0.002, One-Way-ANOVA; Figure 9D). However, the hippocampus innervates the RC to a greater extent than the S (*p* = < 0.001, *t*-test), RSC (*p* = 0.029, Mann-Whitney-*U*-test) and thalamus (*p* = 0.029, Mann-Whitney-*U*-test; Figure 9EIII). Since we injected the RC unilaterally, we are able to differentiate between inputs from ipsi-, and/or contralateral sites (Supplementary Figure S7). We observed a tendency of ipsilateral rather than contralateral cells projecting to the RC from the RSC and DG (Supplementary Figures S7A,B). Interestingly, significantly more ipsilateral cells from the CA3 hippocampus proper (*p* = 0.002, *t*-test) and S (*p* = 0.029, Mann-Whitney-*U*-test) synapse on the RC compared to contralateral cells. However, no eGFP^+^ neurons were seen in the cerebellum.

**Figure 10:**
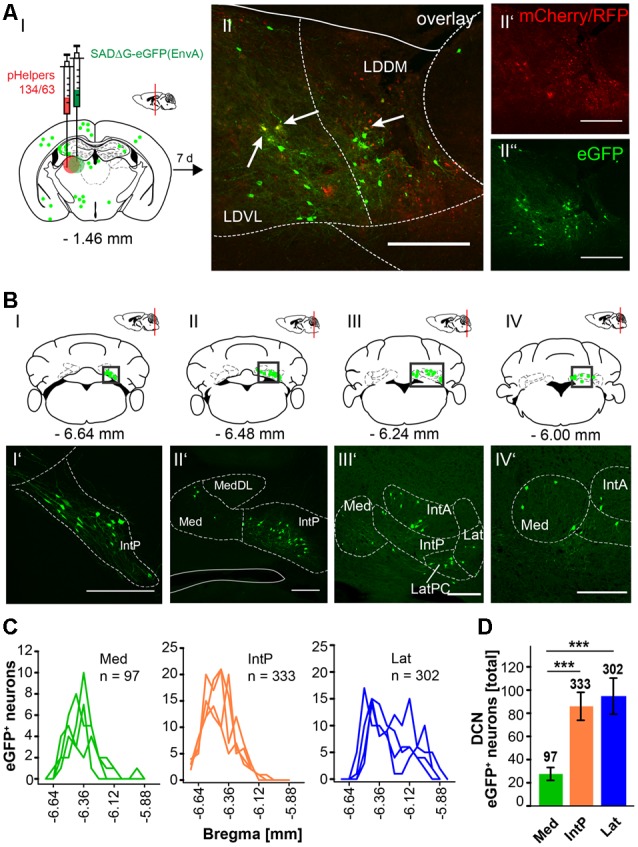
Retrograde modified RABV expression in the laterodorsal thalamus revealed monosynaptic input from the contralateral DCN. **(AI)** Schematic of coronal section at Bregma −1.46 mm and confocal images from an exemplary adult mouse brain injected unilaterally with pHelpers **(AII’)** and rabies virus **(AII”)** in the laterodorsal (dorsomedial and ventrolateral) thalamus (LDDM/LDVL *n* = 4). Green dots represent the retrograde specific, modified RABV tracer expression. The pHelper viruses rAAV8-CBA-RG-mCherry and rAAV8-CBA-mRFP-IRES-TVA (0.2 μl) were injected 7 days prior to the SADΔG-eGFP (EnvA) rabies virus (0.2 μl). Animals were sacrificed 7 days after RABV injection. **(AII)** Double stained neurons were only seen in the desired area as indicated by arrows and indicate starter neurons of retrograde monosynaptic transport. Scale bars: 250 μm. **(BI–IV)** Schematic of coronal brain sections at Bregma −6.64 mm **(BI)**, −6.48 mm **(BII)**, −6.24 mm **(BIII)** and −6.00 mm **(BIV)** depicting eGFP^+^ neurons (green dots) by monosynaptic retrograde transport from the laterodorsal thalamus. Confocal images showing rabies-infected cells in the contralateral cerebellar interpositus (IntP, **BI’–BIV’**), medial (Med, fastigial, **BII’–IV’**) and lateral (Lat, **BIII’,BIV’**) nuclei. Scale bars: 250 μm. **(C)** Line plots mapping the distribution of total eGFP^+^ neurons per mouse by retrograde monosynaptic transport from the LDDM/LDVL to the DCN, medial (green lines), interposed (orange lines) and lateral (blue lines) and their distance to Bregma. **(D)** A total of 333 eGFP^+^ cells were counted for the IntP/IntA and 302 cells were detected in the Lat of all animals, thus showing significantly more input from these two nuclei compared to the Med (97 cells, each *p* ≤ 0.001, One-Way-ANOVA).Significance for comparisons: ***p ≤ 0.001. IntA, anterior interposed cerebellar nucleus; IntP, posterior interposed cerebellar nucleus; Lat, lateral (dentate) cerebellar nucleus; LatPC, parvicellular Lat; LDDM, dorsomedial laterodorsal thalamic nucleus; LDVL, ventrolateral laterodorsal thalamic nucleus; Med, medial (fastigial) cerebellar nucleus; MedDL, dorsolateral protuberance of the Med. The mouse brains in this figure has been reproduced from Franklin and Paxinos (2001).

**Figure 11:**
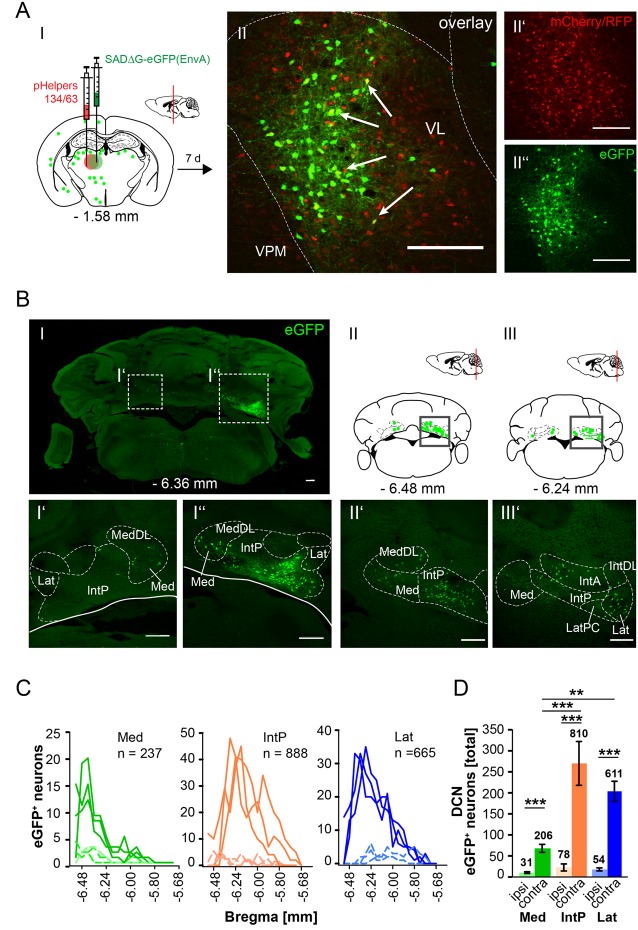
Retrograde, modified RABV expression in the ventrolateral thalamus revealed monosynaptic input from the contralateral and ipsilateral cerebellar nuclei. **(AI)** Schematic of coronal section at Bregma −1.58 mm and confocal image **(AII)** from an exemplary adult mouse brain injected unilaterally with pHelpers **(AII’)** and rabies virus **(AII”)** in the ventrolateral thalamus (VL, *n* = 3). Green dots represent the retrograde specific, modified RABV tracer expression. The pHelper viruses rAAV8-CBA-RG-mCherry and rAAV8-CBA-mRFP-IRES-TVA (1:2 ratio, 0.2 μl) were injected 7 days prior to the SADΔG-eGFP (EnvA) rabies virus (0.2 μl). Mice were sacrificed 7 days after modified RABV injection. **(AII)** Double stained neurons indicate coexpression were only seen in the desired area as indicated by arrows and indicate starter neurons of retrograde monosynaptic transport. Scale bars: 250 μm. **(B)** Confocal image of a coronal brain section at Bregma −6.36 mm **(BI)** showing eGFP^+^ neurons in the DCN. Boxed regions indicate magnified images in **(BI’)** and **(BI”)**. The contralateral cerebellar nucleus interpositus strongly projects to the VL **(BI”)**, supported by weaker input from the ipsi-, and contralateral fastigial nucleus **(BI’–I”)**. The monosynaptic projection from the IntP to the VL resembles a new projection pathway of the cerebellum to the contralateral thalamus, which has not been reported yet. Scale bar: 250 μm. **(BII,BIII)** Schematic of coronal brain sections at Bregma −6.48 mm **(BII)** and −6.24 mm **(BIII)** showing monosynaptic projections from the ipsi-, and contralateral Med **(BII,BIII,BII’)** and contralateral Lat **(BIII’)**. **(C)** Line plots mapping the distribution of total eGFP^+^ neurons per mouse by retrograde monosynaptic transport from the VL to the Med, IntP and Lat, ipsi-, (dashed lines) and contralateral (thick lines) sites. **(D)** Contralateral nuclei generally provide more synaptic input to the VL than ipsilateral nuclei (*p* ≤ 0.001, all *t*-test). However, contralateral IntP and Lat provide more synaptic input to the VL than the Med (for IntP *p* ≤ 0.001; for Lat *p* = 0.006, One-Way-ANOVA).Significance for comparisons: **p ≤ 0.01; ***p ≤ 0.001.IntA, anterior interposed cerebellar nucleus; IntP, posterior interposed cerebellar nucleus; IntDL, dorsolateral hump of IntP; Lat, lateral (dentate) cerebellar nucleus; LatPC, parvicellular Lat; Med, medial (fastigial) cerebellar nucleus; MedDL, dorsolateral protuberance of the medial cerebellar nucleus; VL, ventrolateral thalamic nucleus; VPM, ventral posteromedial thalamic nucleus. The mouse brains in this figure has been reproduced from Franklin and Paxinos (2001).

In this article, we show that LDDM/LDVL and VL in the thalamus is involved in the polysynaptic connections between the cerebellum and hippocampus *via* the S, RSC and RC utilizing both polysynaptic tracers, rAAV8-CMV-WGA-Cre and rAAV8-CMV-TTC-eGFP (Figure 3) and a modified RABV (Figures 7–9). It has been reported that the LDDM participates in spatial learning and memory, while the VL is known to receive fastigial nucleus input in non-human primates (van Groen et al., 2002b; Kelly and Strick, 2003). Based on these data we injected the modified RABV in these thalamic nuclei to identify a potential three-synapse projection pathway from the cerebellum to the hippocampus *via* relay in the thalamus, RSC and/or S and RC. Unilateral injection in the LDDM and LDVL regions of C57/Bl6 mice (*n* ≥ 4) at −1.46 mm from Bregma (Figure 10) revealed co-expression of both helper (mCh/RFP, 0.2 μl) and RABV (eGFP, 0.2 μl) in neurons from both regions (Figure 10AII). We were able to identify a new projection pathway to the LDDM/LDVL from only contralateral areas of the DCN, the medial (fastigial, Med) nucleus (Figures 10BI–IV,C). Neurons of the interpositus nucleus (IntP) and lateral (dentate, Lat) nucleus of the DCN also provide strong monosynaptic input to the LDDM/LDVL (Figures 10B,C). A total of 732 cells were seen in the DCN in all mice (Figure 10D), however, the Med forms less synapses with the LDDM/LDVL than IntP and Lat (*p* ≤ 0.001, One-Way-ANOVA; Figure 10D). Injection of 0.2 μl RABV in the VL at −1.58 mm from Bregma (Figure 11A) revealed co-expressing starter neurons only in the desired area (Figure 11AII). We found both ipsi- and contralateral staining in the DCN (Figure 11BI) that was present through all slices analyzed. The contralateral Lat and IntP of the DCN were identified as a strong synaptic input source to the VL (Figures 11BI”,BII’), but significantly fewer cells were seen in the Med (*p* ≤ 0.001 compared to IntP, One-Way-ANOVA; Figure 11D). In general, the contralateral DCN had more eGFP^+^ neurons than the ipsilateral sites (Figure 11D, each *t*-test). We also found several eGFP^+^ neurons in the pons, equally distributed (Figure 11BI).

**Figure 12:**
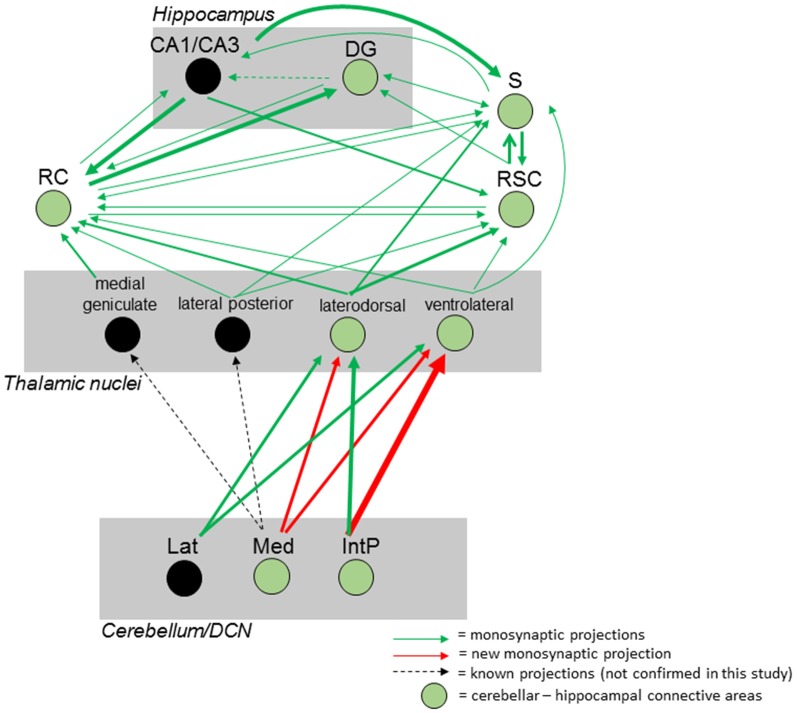
Summary of the mouse cerebellar-hippocampal circuit. Known (black arrows) and observed (green) monosynaptic projections between the cerebellum and hippocampus *via* the thalamus, retrosplenial (RSC) and rhinal (RC) cortices and/or subiculum (S). A step by step injection of rabies virus started in the DG confirmed monosynaptic input from S, RC and RSC. While the S projects to and receives input from CA1/CA3 and DG, RSC, and RC, monosynaptic input was confirmed from the laterodorsal, ventrolateral and lateral posterior thalamic nuclei. Injections in the RSC confirmed monosynaptic input from these thalamic nuclei, too. By injecting these thalamic nuclei, new monosynaptic projections (red arrows) from the medial cerebellar nucleus to the laterodorsal and ventrolateral thalamus, as well as a new monosynaptic projection from the posterior interpositus cerebellar nucleus to the ventrolateral thalamus were identified in this study. Injecting the RC confirmed additional monosynaptic input from the medial geniculate thalamic nucleus which was not injected with rabies and can only be hypothesized to connect the cerebellum with the hippocampus (black circles). Thickness of arrows indicate strength of observed projections. IntP, posterior interposed cerebellar nucleus; Lat, lateral (dentate) cerebellar nucleus; Med, medial (fastigial) cerebellar nucleus; DG, dentate gyrus; S, subiculum; RC, rhinal cortex; RSC, retrosplenial cortex.

Taken together, we were able to identify a polysynaptic cerebellar-hippocampal connection by use of monosynaptic retrograde and polysynaptic anterograde and retrograde virus-based tracing. We found a new projection pathway from the medial cerebellar nucleus to the laterodorsal thalamic nuclei, which has not been reported. We also found that the Med projects to the ventrolateral thalamic nuclei in mice, as well as the interpositus cerebellar nuclei synapse on laterodorsal and ventrolateral thalamic nuclei which has been described partially in other species. Both thalamic nuclei synapse on either the subiculum, rhinal cortex and RSC may communicate *via* direct monosynaptic connections, as well as projecting to the hippocampus (Figure 12).

## Discussion

The cerebellum assists in spatial navigation by participating in building the hippocampal spatial map (Rochefort et al., 2011). The vestibular system plays a vital role in stabilizing gaze during head movements in addition to controlling posture and spontaneous reflexes. Impairments in vestibular inputs diminish learning and memory in particular spatial learning by affecting the proper function of head direction, place and grid cells. Moreover, loss of the vestibular system leads to degeneration of the hippocampus and its dendritic branches and impaired spatial memory in humans (Brandt et al., 2005; Smith et al., 2005; Cronin et al., 2017). Consequently, the cerebellum must synaptically communicate with the hippocampus, either directly or indirectly through other brain regions, possibly involved in navigation such as the subiculum (S) or RSC. Previous studies already showed a functional connectivity between cerebellum and hippocampus in both human and mice (Fischer et al., 2008; O’Reilly et al., 2010; Iglói et al., 2015; Onuki et al., 2015; Watson et al., 2019), but the identification of a neuronal pathway remains to be determined. To improve our understanding of how the cerebellum synaptically communicates with the hippocampus, we used both polysynaptic anterograde and retrograde and monosynaptic retrograde virus-based approaches. Based on our tracing results, we here propose a polysynaptic circuitry from cerebellar fastigial nucleus (Med) with a relay in the LDDM/LDVL and VL, which in turn synapses on S, RC and RSC, which all project to the hippocampus (Figure 12). We, however, failed to identify a monosynaptic projection between cerebellum and hippocampus. This seems reasonable since the cerebellum has been shown to connect with the cerebral cortex *via* only polysynaptic circuits in primates (Evarts and Thach, 1969; Middleton and Strick, 1994, 2001; Kelly and Strick, 2003). Moreover, the involvement of the laterodorsal and ventrolateral thalamic nuclei has been confirmed in other studies, as each known cerebellar projection synapses onto the thalamus, that functions as a relay (Buckner et al., 2011).

Both tracer viruses, modified RABV and rAAV8-CMV-WGA-Cre/rAAV8-CMV-TTC-eGFP failed to identify a monosynaptic projection from the cerebellum to the hippocampus. However, we cannot exclude the possibility that a weak monosynaptic transneuronal connection between both structures exist, which is not detectable with our tracing methods. In agreement with our findings, tracing studies using rabies virus as a retrograde, polysynaptic, transneuronal tracer in the dentate gyrus of the hippocampus, found a multisynaptic pathway to restricted regions of the cerebellum which include lobules VI/VII, Crus I, lobule IX and paraflocculus (Watson et al., 2019). Additionally, they performed cerebello-hippocampal LFP coherence recordings in combination with spatial navigation tests in mice to confirm the synchronization of LFP activity between CrusI and the dorsal hippocampus (DG) during these tasks. In the 80s tracing studies using polysynaptic radiolabeled amino acids or wheat germ agglutinin conjugated to horseradish peroxidase (WGA-HRP) reported a transient direct projection from the cerebral cortex to the DCN and/or cerebellar cortex in young kittens, rabbit fetuses and in pouch young North American opossum (Tolbert and Panneton, 1983, 1984; Panneton and Tolbert, 1984; Cabana and Martin, 1986; Tolbert, 1989a,b). In addition transient hippocampal projections to the cerebellum in chicken, from areas of the hippocampal formation project to lobules VI–VIII in young but not adult animals were observed (Liu et al., 2012). Direct cerebrocerebellar projections have also been reported in zebra finches and rats (Wild and Williams, 2000), however, they were sparse and temporary. Note that most of these studies used radiolabeled amino acids or WGA-HRP tracers, which cannot distinguish between mono- and polysynaptic connections thus, the interpretation of these results are error-prone.

In the last decade, more advanced WGA tracing tools have been developed combining the CRE and rAAV systems to optimize the specificity and expression of the WGA tracer in the brain (Jarvik et al., 1981). Despite these advances, the interpretation of the data is limited. For example the transduction of rAAV tagged with a fluorescent protein show conflicting reports in the literature, depending on the serotype, region of interest or titer used (Ohta et al., 2011; Espallergues et al., 2012). Moreover the serotype AAV8 used in this study was previously demonstrated to have a higher efficacy to infect hippocampal and cerebellar neurons (Heinemann et al., 1991; Broekman et al., 2006), but in addition has been observed to transport minimally in the retrograde direction *via* axonal terminals (Hastings et al., 1981; Carlson et al., 2016). Similarly, WGA has also been reported to be bidirectional depending on the serotype and brain region, although it has a preference for anterograde transport (Whitney et al., 2016). In this study, we used a rAAV-WGA-Cre vector, which transduced in the cerebellar and hippocampal regions. However, due to the high expression levels needed of WGA-Cre in transduced cells for transneuronal labeling over multiple synapses, long incubation times were required (Hendricks et al., 2003). In order to circumvent these bidirectional, polysynaptic pitfalls using the rAAV-WGA-Cre system, we implemented and confirmed the initial connections using the AAV system with the deletion-mutant rabies virus system. The advantages of this system are that one can track not only the injection site and spread of the virus but also monosynaptic, retrograde connections. However, we are limited to the interpretation of our results with trisynaptic connections (A to B to C). For example, we can determine a projection from A to B but not with certainty A to B to C because, we cannot assume that A is connected to C *via* B. The cells traced in B may be connected with other local cells in B expressing the rabies virus, which receive input from cells in C. Moreover, the rAAV helper viruses could be presynaptically transferred from axons projecting into the injection site. To control for this, we screened for mCherry/RFP fluorescence outside the injection site and found that only in one case (Supplementary Figure S6B), red fluorescence was seen in a synaptically connected area. However, no eGFP^+^ cells were observed due to the high expression of glycoprotein and TVA receptor required for RABV transcomplementation (Ugolini, 1995; Kelly and Strick, 2000; Wickersham et al., 2007a,b).

We describe here the first tracing study between the cerebellum and hippocampus in mice using a mono-transsynaptic, retrograde tracer system based on a modified rabies virus and well-established polysynaptic tracers rAAV8-CMV-WGA-Cre and rAAV8-CMV-TTC-eGFP (summarized in Figure 12). Our observations strengthen the notion of a polysynaptic circuitry between the cerebellum and hippocampus, that utilizes the thalamus as a relay center to cortical areas as first described in monkeys by the Strick lab (Middleton and Strick, 1994). This and work from others suggests that the RSC connects to the cerebellum with the hippocampus *via* polysynaptic circuits as it receives projections from the vestibular nuclei in the pons *via* the lateral thalamic nuclei which have been shown to receive cerebellar input (Sripanidkulchai and Wyss, 1986; Middleton and Strick, 1994, 2001; Rochefort et al., 2013). The injection of rAAV8-CMV-WGA-Cre in the RSC resulted in a strong staining, predominately in the thalamus, including the dorsomedial laterodorsal thalamus (LDDM), anteroventral thalamic nucleus and other areas (Figure 3). This is not surprising, since these two nuclei have been reported to project to RSA and RSG in rats and receive projections from the RSC (Sripanidkulchai and Wyss, 1986; Vann et al., 2009). Injections of the WGA-Cre tracer in the DCN (Figure 1) and DG (Figure 2) only resulted in little to no cells expressing tdTomato in the thalamus, instead more neurites were seen crossing these thalamic nuclei. Both the LDDM and ventrolateral (LDVL) thalamic nuclei participate in spatial learning and memory but there is no projection from the cerebellum reported (van Groen et al., 2002a). rAAV8-CMV-WGA-Cre injections in the RSA stained the LDDM and cerebellar lobules IV, V, VI, VIII and X, as well as right PFl, CrusI, CrusII and left simple lobule, suggesting a cerebellar connection to the LDDM. Since the LDDM and LDVL are known to project to the RSC, which in turn projects to the subiculum and dentate gyrus, the RSA might serve as relay between the cerebellum and the hippocampus (van Groen and Wyss, 1990, 2003; Wyss and Van Groen, 1992; Aggleton et al., 2014). The same principle may be applied to the subiculum, which was shown in this study, but also by other scientists to project to the DG and RSC (Hartley et al., 2013; Sun et al., 2014) and receives input from the LDDM/LDVL. Thus, the subiculum might also serve as a linker between cerebellum and hippocampus *via* the laterodorsal thalamic nucleus.

Additional injections with our mono-transsynaptic RABV in the RSC (Figure 8), rhinal cortex (Figure 9) and subiculum (Figure 7) demonstrated projections from the laterodorsal medial and ventral (LDDM, LDVL) thalamic nuclei. These lateral posterior regions of the thalamus are known to connect with the fastigial nucleus. Furthermore recently published work by Rondi-Reig’s lab confirmed a polysynaptic cerebello-hippocampal pathway both anatomically and functionally, implementing a CrusI/fastigial nucleus/dentate gyrus pathway important for spatial navigation in mice (Pearlstein et al., 2011; Watson et al., 2019). Thus, injecting the laterodorsal (Figure 10) thalamic nuclei, which are involved in spatial learning and memory (van Groen et al., 2002b) revealed innervation of the contralateral cerebellar interpositus and fastigial nucleus (Figure 10), which has not been reported before (van Groen et al., 2002a). The ventrolateral thalamic nucleus (VL) was stained after modified RABV injection in the RSC (Figure 8), S (Figure 7) and RC (Figure 9) and although there were only a few neurons expressing eGFP, they verify monosynaptic projections from the VL to these areas, which is involved in spatial navigation (Alexander and Nitz, 2015; Chrastil et al., 2015). The VL was shown to receive projections from the fastigial nucleus in non-human primates and shown to serve as a relay of these axons to the primary motor cortex (Kelly and Strick, 2003). Moreover lesion of the FN resulted in degenerated hippocampal fibers in different species suggesting a FN projection to the hippocampus of unknown relay (Harper and Heath, 1973; Heath and Harper, 1974). Injection of modified rabies virus in the VL (Figure 11) revealed contralateral innervation from all DCN, but also ipsilateral input from the fastigial nucleus. Thus, we were able to confirm fastigial nucleus input to the hippocampus with a relay in the ventrolateral thalamus.

Surprisingly, we did not observe tdTomato^+^ cells in the LDDM/LDVL or the VL following rAAV-WGA-Cre injections in the DCN. Instead, we found a few cells in the lateral posterior thalamic nuclei. Moreover, rAAV-WGA-Cre injections in the DCN revealed the most tdTomato expression in the medial cerebellar nucleus. However RABV injections in the LDDM/LDLV (Figure 10) and VL (Figure 11) showed that the medial cerebellar nucleus provides significantly less input to these regions than interposed or lateral cerebellar nucleus. This may explain why we did not see tdTomato expression after rAAV-WGA-Cre injection in the DCN. The projection from the cerebellum to the hippocampus proposed by our data is mostly based on step-by-step retrograde monosynaptic transport, which may differ from an anterograde hippocampal-cerebellar pathway.

Several studies suggested cerebellar participation in spatial navigation (Rochefort et al., 2011; Iglói et al., 2015; Onuki et al., 2015). Although these studies support cerebellar involvement in spatial navigation, a direct neuronal projection pathway is still elusive. We here present a tracing study in mice that shows a cerebellar-hippocampal polysynaptic projection pathway *via* the laterodorsal and ventrolateral thalamus to RSC, subiculum and rhinal cortex. We were able to show new projections from the cerebellar interpositus and fastigial nucleus to contralateral LDDM/LDVL and VL, but also ipsilateral projections from the cerebellar fastigial nucleus to the VL. In contrast to Watson et al. (2019), who found retrogradely-labeled rabies-infected cells mostly in the dentate and fastigial nuclei, we here report monosynaptic input from mostly interpositus and dentate nuclei to LDDM/LDVL and VL, with 3–4 times fewer cells in the fastigial nucleus. However, our results further strengthen the notion of a cerebellar participation in hippocampal-based spatial navigation processing, however functional studies to confirm this polysynaptic connection needs to be investigated.

## Ethics Statement

The present study was carried out in accordance with the European Communities Council Directive of 2010 (2010/63/EU) for care of laboratory animals and approved by a local ethics committee (Bezirksamt Arnsberg) and the animal care committee of North Rhine-Westphalia, Germany, based at the LANUV (Landesamt für Umweltschutz, Naturschutz und Verbraucherschutz, Nordrhein-Westfalen, D-45659 Recklinghausen, Germany). The study was supervised by the animal welfare commission of the Ruhr-University Bochum. All efforts were made to minimize the number of mice used for this study.

## Author Contributions

MM and SH conceived and designed the experiments. PB and MM performed tracing studies, rAAV virus production and tracing vector design performed by PB and MM. Modified rabies virus provided by MS. MM, PB, SH and MS wrote the manuscript.

## Conflict of Interest Statement

The authors declare that the research was conducted in the absence of any commercial or financial relationships that could be construed as a potential conflict of interest.

## Supplementary Material

FIGURE S1 Additional polysynaptic targets observed after injection of the polysynaptic anterograde tracer AAV8-CMV-WGA-Cre in the DCN of tdTomato^+^ mice. **(A)** Example single tdTomato^+^ cell bodies were imaged from the dorsal raphe nuclei [dorsal part (DRD), ventral (DRV), ventrolateral (DRVL), the raphe cap (RC) and ventrolateral periaqueducal gray (VLPAG)] and the lateral lemniscus, dorsal (DLL) and intermediate nucleus (ILL; **A’**) at −4.60 mm from Bregma. Scale bars: 250 μm. **(B)** Example single tdTomato^+^ cell bodies were imaged from the magnocellular red nucleus (RMC) and superior cerebellar peduncle (scp) and right dorsal and ventral medial geniculate nucleus (MGD, MGV; **B’**) at −3.80 mm from Bregma. tdTomato^+^ neurites were additionally seen in the posterior intralaminar thalamic nucleus (PIL), suprageniculate thalamic nucleus (SG) and the medial part of the medial geniculate nucleus (MGM). Scale bars: 250 μm **(C)** Example single tdTomato^+^ cell bodies imaged from the prerubral field (PRl; **C**) and lateral secondary visual cortex (V2L; **C’**) at −2.54 mm from Bregma. Scale bars: 250 μm. **(D)** Polysynaptically tdTomato^+^ cells at 2.58 mm from Bregma seen in the granular cell layer of the olfactory bulb (GrO) and internal plexiform layer of the olfactory bulb (IPI) and frontal association cortex (FrA; **D’**) Scale bars: 250 μm. The mouse brains in this figure has been reproduced from Franklin and Paxinos (2001).

FIGURE S2 Additional polysynaptic targets observed after injection of the polysynaptic anterograde tracer AAV8-CMV-WGA-Cre in the dentate gyrus of tdTomato^+^ mice. **(A)** Polysynaptic tdTomato^+^ cells at −4.36 mm from Bregma in the external cortex of the inferior colliculus (ECIC), nucleus of the brachium of the inferior colliculus (BIC) and the brachium colliculus (bic). **(A’)** tdTomato^+^ neurites in the lateral lemniscus, intermediate (ILL) and ventral nuclei (VLL). Scale bar: 100 μm. **(A”)** tdTomato^+^ cell bodies and neurites in the tectospinal tract (ts), median raphe nucleus (MnR) and paramedian raphe nucleus (PMnR) and the reticulotegmental nucleus of the pons (RtTg). Scale bars: 100 μm. **(B)** Exemplary tdTomato^+^ neurites in the medial geniculate nucleus, dorsal (MGD), ventral (MGV) and medial (MGM) parts and the suprageniculate thalamic nucleus (SG) at −3.28 mm from Bregma. Scale bar: 100 μm **(C)** Exemplary tdTomato^+^ neurites in the lateral posterior thalamic nucleus, mediorostral (LPMR) and laterorostral parts (LPLR). **(C’)** tdTomato^+^ cell bodies in the secondary visual cortex, mediolateral area (V2ML). Scale bars 100 μm. **(D)** At 0.74 mm from Bregma, tdTomato^+^ cell bodies were seen in the medial septal nucleus (MS) and in the nucleus of the vertical limb of the diagonal band (VDB). Scale bar: 250 μm. **(D’)** Intense tdTomato^+^ neurites in the lambdoid septal zone (Ld) and dorsal (LSD) and intermediate nuclei of the lateral septal nucleus (LSI). Scale bars: 250 μm. The mouse brains in this figure has been reproduced from Franklin and Paxinos (2001).

FIGURE S3 Additional monosynaptic connected areas observed after injection of the retrograde tracer SADΔG-eGFP in the Hippocampus of C57/Bl6 mice. **(A)** Coronal brain section at −3.88 mm from Bregma depicting an eGFP^+^ neuron (green dots) in the rostral linear nucleus of the raphe (RLi) and interpeduncular nucleus, rostral subnucleus (IPR, **A’**) by retrograde monosynaptic transport from the hippocampus. Scale bar: 100 μm. **(B)** Confocal image from boxed area depicting eGFP^+^ cell bodies in the medial and lateral supramammillary nucleus (SuML, SuMM) at −2.70 mm from Bregma. Scale bar: 500 μm. **(C)** Confocal image of eGFP^+^ neurons in the nucleus of the horizontal limb of the diagonal band (HDB) at – 0.34 mm from Bregma. Scale bar: 200 μm. **(D)** Confocal image of a multiple eGFP^+^ cell bodies in the medial septal nucleus (MS) and medial preoptic area (MPA). Scale bar: 250 μm. The mouse brains in this figure has been reproduced from Franklin and Paxinos (2001).

FIGURE S4 Additional monosynaptic connected areas observed after injection of the retrograde tracer SADΔG-eGFP in the Dentate Gyrus of C57/Bl6 mice. **(A)** Coronal brain section at −6.12 mm from Bregma depicting a eGFP^+^ neuron (green dots) in the alpha part of the gigantocellular reticular nucleus (GiA) by retrograde monosynaptic transport from the dentate gyrus (DG). Scale bar: 250 μm. **(B)** Confocal image from boxed area depicting a single eGFP^+^ cell body in the ventrolateral periaqueductal gray (VLPAG) at −4.72 mm from Bregma. Scale bar: 250 μm. **(C)** Confocal image of eGFP^+^ neurons in the lateral supramammillary nucleus (SuML) at −2.80 mm from Bregma. Scale bar: 250 μm. **(D)** Confocal image of a multiple eGFP^+^ cell bodies in the nucleus of the ventral limb of the diagonal band (VDB). Scale bar: 250 μm. The mouse brains in this figure has been reproduced from Franklin and Paxinos (2001).

FIGURE S5 Additional monosynaptic connected areas observed after injection of the retrograde tracer SADΔG-eGFP in the retrosplenial cortex of C57/Bl6 mice. **(A)** Coronal brain section at −5.68 mm from Bregma depicting a eGFP^+^ neuron (green dots) in the pontine reticular nucleus (PnC) by retrograde monosynaptic transport from the retrosplenial cortex (RC). Scale bar: 100 μm. **(B,B’)** Confocal images from boxed areas depicting eGFP^+^ cell bodies in the medial raphe nucleus (MnR; **B**, Scale bar: 250 μm) and B9 serotonergic cells **(B’**, Scale bar: 100 μm) at −4.48 mm from Bregma**. (C)** Confocal image of eGFP^+^ neurons in the lateral supramammillary nucleus (SuML) at −3.16 mm from Bregma. Scale bar: 250 μm. **(D)** Confocal image of a single rabies-infected cell in the secondary auditory cortex (AuD). Scale bar: 100 μm. The mouse brains in this figure has been reproduced from Franklin and Paxinos (2001).

FIGURE S6 Additional monosynaptic connected areas observed after injection of the retrograde tracer SADΔG-eGFP in the rhinal cortex of C57/Bl6 mice. **(A)** Confocal image showing higher magnification of a eGFP^+^ neuron in the Barrington’s nucleus (Bar) in the pons at −5.52 mm from Bregma. Scale bar: 250 μm **(A’)** Single eGFP^+^ cell in the alpha part of the gigantocellular reticular nucleus (GiA) in the pons at −5.52 mm from Bregma. Scale bar: 250 μm. **(B)** Several pyramidal cell-like neurons in the CA1 region and pyramidal cell layer (Py) at −3.80 mm from Bregma on the ipsilateral side. Scale bar: 250 μm. **(B’)** Only one cell in the Py was seen on the contralateral side. Scale bar: 250 μm. **(B”)** Confocal image showing several eGFP^+^ cells in the dorsal (MGD) and ventral (MGV) parts of medial geniculate nucleus at −3.40 mm from Bregma. Scale bar: scale bar: 250 μm. **(C)** Brain scheme at −1.82 mm. Green dots represent eGFP^+^ cells in all analyzed mice in the RSC, ipsilateral CA1 and CA2. High magnification image showing one representative single eGFP^+^ neuron found in the ipsilateral mediorostral lateral posterior (LPMR) of the thalamus. Scale bar: 250 μm **(D)** Brain scheme at 0.86 mm from Bregma showing representative summarized eGFP^+^ cells. Squared boxes represent areas of confocal images of the ipsilateral secondary motor cortex (M2, **D’**), ipsilateral nucleus of the horizontal limb (HDB, **D”**) and medial septal nucleus (MS) and lambdoid zone (Ld; **D”’**). Scale bars: 250 μm. The mouse brains in this figure has been reproduced from Franklin and Paxinos (2001).

FIGURE S7 Additional statistical analysis of all eGFP^+^ neurons observed in different regions on the murine brain after injection of the retrograde tracer SADΔG-eGFP in the rhinal cortex of C57/Bl6 mice. **(A)** eGFP^+^ cells that were found in the RSC at different mm from Bregma. The four traces represent the analyzed *n* = 4 mice. **(AI)** A total of 275 eGFP^+^ cells were found in the ipsilateral RSC, while only 76 cells were found in the contralateral RSC **(AII)**. **(AIII)** No statistical difference was found between ipsi., and contralateral sites. *T*-test, *p* = 0.069. **(B)** Summary of eGFP^+^ cells that were found in the DG and CA3 at different mm from Bregma. The four traces represent the analyzed *n* = 4 mice. **(BI)** A total of 207 eGFP^+^ cells were found in the ipsilateral DG, while 145 cells were found in the contralateral DG. No statistical difference was found between sites. *T*-test, *p* = 0.249 **(BIII)**. **(BII)** Traces of all eGFP^+^ cells observed in the ipsilateral (orange thick lines, 2,472 cells) and contralateral (dashed lines, 698) CA3 region of the hippocampus. Three-thousand one-hundred and seventy cells were found in all analyzed mice, with significantly more input on the RC from the ipsilateral CA3 cells (*t*-test, *p* = 0.002; **BIII)**. **(BIII)** A total of 28,082 eGFP^+^ cells were found in the CA1, with only eight cells found on the contralateral site (data not shown). Significantly more cells from the CA1 region are connected to the RC when compared to all CA3 cells (*t*-test, *p* = ≤ 0.001) and all cells from the DG (*t*-test, *p* = 0.029). **(C)** Total eGFP^+^ neurons observed in the subiculum. Significantly more cells from the ipsilateral S (4,233) compared to the contralateral S (36) provide input to the RC (*t*-test, *p* = 0.029). Significance for comparisons: ns not significant; **p* ≤ 0.05; ***p* ≤ 0.01; ****p* ≤ 0.001.

## References

[B2] Aggleton J. P. Saunders R. C. Wright N. F. Vann S. D. (2014). The origin of projections from the posterior cingulate and retrosplenial cortices to the anterior, medial dorsal and laterodorsal thalamic nuclei of macaque monkeys. Eur. J. Neurosci. 39, 107–123. 10.1111/ejn.12389 24134130 PMC4112842

[B3] Agster K. L. Burwell R. D. (2013). Hippocampal and subicular efferents and afferents of the perirhinal, postrhinal, and entorhinal cortices of the rat. Behav. Brain Res. 254, 50–64. 10.1016/j.bbr.2013.07.005 23872326 PMC3792719

[B4] Alexander A. S. Nitz D. A. (2015). Retrosplenial cortex maps the conjunction of internal and external spaces. Nat. Neurosci. 18, 1143–1151. 10.1038/nn.4058 26147532

[B5] Baillieux H. De Smet H. J. Paquier P. F. De Deyn P. P. Marien P. (2008a). Cerebellar neurocognition: insights into the bottom of the brain. Clin. Neurol. Neurosurg. 110, 763–773. 10.1016/j.clineuro.2008.05.013 18602745

[B6] Baillieux H. Verslegers W. Paquier P. De Deyn P. P. Marien P. (2008b). Cerebellar cognitive affective syndrome associated with topiramate. Clin. Neurol. Neurosurg. 110, 496–499. 10.1016/j.clineuro.2008.01.003 18304728

[B7] Brandt T. Schautzer F. Hamilton D. A. Bruning R. Markowitsch H. J. Kalla R.. (2005). Vestibular loss causes hippocampal atrophy and impaired spatial memory in humans. Brain 128, 2732–2741. 10.1093/brain/awh617 16141283

[B8] Broekman M. L. Comer L. A. Hyman B. T. Sena-Esteves M. (2006). Adeno-associated virus vectors serotyped with AAV8 capsid are more efficient than AAV-1 or -2 serotypes for widespread gene delivery to the neonatal mouse brain. Neuroscience 138, 501–510. 10.1016/j.neuroscience.2005.11.057 16414198

[B9] Buckner R. L. Krienen F. M. Castellanos A. Diaz J. C. Yeo B. T. (2011). The organization of the human cerebellum estimated by intrinsic functional connectivity. J. Neurophysiol. 106, 2322–2345. 10.1152/jn.00339.2011 21795627 PMC3214121

[B10] Büning H. Perabo L. Coutelle O. Quadt-Humme S. Hallek M. (2008). Recent developments in adeno-associated virus vector technology. J. Gene Med. 10, 717–733. 10.1002/jgm.1205 18452237

[B11] Burguière E. Arabo A. Jarlier F. De Zeeuw C. I. Rondi-Reig L. (2010). Role of the cerebellar cortex in conditioned goal-directed behavior. J. Neurosci. 30, 13265–13271. 10.1523/jneurosci.2190-10.2010 20926652 PMC6634747

[B12] Cabana T. Martin G. F. (1986). Development of projections from somatic motor-sensory areas of neocortex to the diencephalon and brainstem in the North American opossum. J. Comp. Neurol. 251, 506–516. 10.1002/cne.902510406 2431011

[B13] Carlson K. S. Whitney M. S. Gadziola M. A. Deneris E. S. Wesson D. W. (2016). Preservation of essential odor-guided behaviors and odor-based reversal learning after targeting adult brain serotonin synthesis. eNeuro 3:ENEURO.0257-16.2016. 10.1523/eneuro.0257-16.2016 27896310 PMC5112565

[B14] Chamberlin N. L. Du B. de Lacalle S. Saper C. B. (1998). Recombinant adeno-associated virus vector: use for transgene expression and anterograde tract tracing in the CNS. Brain Res. 793, 169–175. 10.1016/s0006-8993(98)00169-3 9630611 PMC4961038

[B15] Chrastil E. R. Sherrill K. R. Hasselmo M. E. Stern C. E. (2015). There and back again: hippocampus and retrosplenial cortex track homing distance during human path integration. J. Neurosci. 35, 15442–15452. 10.1523/jneurosci.1209-15.2015 26586830 PMC6605486

[B16] Colombel C. Lalonde R. Caston J. (2004). The effects of unilateral removal of the cerebellar hemispheres on spatial learning and memory in rats. Brain Res. 1004, 108–115. 10.1016/j.brainres.2003.10.075 15033425

[B17] Cronin T. Arshad Q. Seemungal B. M. (2017). Vestibular deficits in neurodegenerative disorders: balance, dizziness, and spatial disorientation. Front. Neurol. 8:538. 10.3389/fneur.2017.00538 29123498 PMC5662638

[B18] Deneris E. S. Wyler S. C. (2012). Serotonergic transcriptional networks and potential importance to mental health. Nat. Neurosci. 15, 519–527. 10.1038/nn.3039 22366757 PMC3594782

[B19] Dum R. P. Strick P. L. (2003). An unfolded map of the cerebellar dentate nucleus and its projections to the cerebral cortex. J. Neurophysiol. 89, 634–639. 10.1152/jn.00626.2002 12522208

[B20] Espallergues J. Teegarden S. L. Veerakumar A. Boulden J. Challis C. Jochems J.. (2012). HDAC6 regulates glucocorticoid receptor signaling in serotonin pathways with critical impact on stress resilience. J. Neurosci. 32, 4400–4416. 10.1523/jneurosci.5634-11.2012 22457490 PMC3355377

[B21] Evarts E. V. Thach W. T. (1969). Motor mechanisms of the CNS: cerebrocerebellar interrelations. Annu. Rev. Physiol. 31, 451–498. 10.1146/annurev.ph.31.030169.002315 4885774

[B1] Fischer A. H. van der Loo B. Shär M. G. Zbinden R. Duru F. Brunckhorst C.. (2008). Serological evidence for the association of Bartonella henselae infection with arrhythmogenic right ventricular cardiomyopathy. Clin. Cardiol. 31, 469–471. 10.1002/clc.20269 18666174 PMC6653530

[B200] Franklin K. B. J. Paxinos G. (2001). The Mouse Brain in Stereotaxic Coordinates. 2nd Edn. San Diego: Academic Press.

[B2300] Grieger J. C. Choi V. M. Samulski R. J. (2006). Production and characterization of adeno-associated viral vectors. Nat. Protoc. 1, 1412–1428. 10.1038/nprot.2006.207 17406430

[B22] Harper J. W. Heath R. G. (1973). Anatomic connections of the fastigial nucleus to the rostral forebrain in the cat. Exp. Neurol. 39, 285–292. 10.1016/0014-4886(73)90231-8 4573973

[B23] Hartley T. Lever C. Burgess N. O’Keefe J. (2013). Space in the brain: how the hippocampal formation supports spatial cognition. Philos. Trans. R. Soc. Lond. B Biol. Sci. 369:20120510. 10.1098/rstb.2012.0510 24366125 PMC3866435

[B24] Hastings W. L. Aaron J. L. Deneris J. Kessler T. R. Pons A. B. Razzeca K. J.. (1981). A retrospective study of nine calves surviving five months on the pneumatic total artificial heart. Trans. Am. Soc. Artif. Intern. Organs 27, 71–76. 7331162

[B25] Heath R. G. Harper J. W. (1974). Ascending projections of the cerebellar fastigial nucleus to the hippocampus, amygdala and other temporal lobe sites: evoked potential and histological studies in monkeys and cats. Exp. Neurol. 45, 268–287. 10.1016/0014-4886(74)90118-6 4422320

[B26] Heinemann S. F. Boulter J. Connolly J. Deneris E. Duvoisin R. Hartley M.. (1991). Brain nicotinic receptor genes. NIDA Res. Monogr. 111, 3–23. 1775184

[B27] Hendricks T. J. Fyodorov D. V. Wegman L. J. Lelutiu N. B. Pehek E. A. Yamamoto B.. (2003). Pet-1 ETS gene plays a critical role in 5-HT neuron development and is required for normal anxiety-like and aggressive behavior. Neuron 37, 233–247. 10.1016/s0896-6273(02)01167-4 12546819

[B28] Iglói K. Doeller C. F. Paradis A. L. Benchenane K. Berthoz A. Burgess N.. (2015). Interaction between hippocampus and cerebellum crus I in sequence-based but not place-based navigation. Cereb. Cortex 25, 4146–4154. 10.1093/cercor/bhu132 24947462 PMC4886832

[B29] Jarvik R. K. Kessler T. R. McGill L. D. Olsen D. B. DeVries W. C. Deneris J.. (1981). Determinants of pannus formation in long-surviving artificial heart calves, and its prevention. Trans. Am. Soc. Artif. Intern. Organs 27, 90–96. 7331167

[B30] Kaplitt M. G. Leone P. Samulski R. J. Xiao X. Pfaff D. W. O’Malley K. L.. (1994). Long-term gene expression and phenotypic correction using adeno-associated virus vectors in the mammalian brain. Nat. Genet. 8, 148–154. 10.1038/ng1094-148 7842013

[B31] Kelly R. M. Strick P. L. (2000). Rabies as a transneuronal tracer of circuits in the central nervous system. J. Neurosci. Methods 103, 63–71. 10.1016/s0165-0270(00)00296-x 11074096

[B32] Kelly R. M. Strick P. L. (2003). Cerebellar loops with motor cortex and prefrontal cortex of a nonhuman primate. J. Neurosci. 23, 8432–8444. 10.1523/JNEUROSCI.23-23-08432.2003 12968006 PMC6740694

[B33] Kwon I. Schaffer D. V. (2008). Designer gene delivery vectors: molecular engineering and evolution of adeno-associated viral vectors for enhanced gene transfer. Pharm. Res. 25, 489–499. 10.1007/s11095-007-9431-0 17763830 PMC2265771

[B34] Liu W. Zhang Y. Yuan W. Wang J. Li S. (2012). A direct hippocampo-cerebellar projection in chicken. Anat. Rec. 295, 1311–1320. 10.1002/ar.22515 22692931

[B35] Madisen L. Zwingman T. A. Sunkin S. M. Oh S. W. Zariwala H. A. Gu H.. (2010). A robust and high-throughput Cre reporting and characterization system for the whole mouse brain. Nat. Neurosci. 13, 133–140. 10.1038/nn.2467 20023653 PMC2840225

[B36] Middleton F. A. Strick P. L. (1994). Anatomical evidence for cerebellar and basal ganglia involvement in higher cognitive function. Science 266, 458–461. 10.1126/science.7939688 7939688

[B37] Middleton F. A. Strick P. L. (2001). Cerebellar projections to the prefrontal cortex of the primate. J. Neurosci. 21, 700–712. 10.1523/JNEUROSCI.21-02-00700.2001 11160449 PMC6763818

[B38] Molinari M. Chiricozzi F. R. Clausi S. Tedesco A. M. De Lisa M. Leggio M. G. (2008). Cerebellum and detection of sequences, from perception to cognition. Cerebellum 7, 611–615. 10.1007/s12311-008-0060-x 18941861

[B39] Niedworok C. J. Schwarz I. Ledderose J. Giese G. Conzelmann K. K. Schwarz M. K. (2012). Charting monosynaptic connectivity maps by two-color light-sheet fluorescence microscopy. Cell Rep. 2, 1375–1386. 10.1016/j.celrep.2012.10.008 23142666

[B40] Ohta Y. Kosaka Y. Kishimoto N. Wang J. Smith S. B. Honig G.. (2011). Convergence of the insulin and serotonin programs in the pancreatic β-cell. Diabetes 60, 3208–3216. 10.2337/db10-1192 22013016 PMC3219954

[B41] Onuki Y. Van Someren E. J. De Zeeuw C. I. Van der Werf Y. D. (2015). Hippocampal-cerebellar interaction during spatio-temporal prediction. Cereb. Cortex 25, 313–321. 10.1093/cercor/bht221 23968839

[B42] O’Reilly J. X. Beckmann C. F. Tomassini V. Ramnani N. Johansen-Berg H. (2010). Distinct and overlapping functional zones in the cerebellum defined by resting state functional connectivity. Cereb. Cortex 20, 953–965. 10.1093/cercor/bhp157 19684249 PMC2837094

[B43] Panneton W. M. Tolbert D. L. (1984). The collateral origin of a transient cerebrocerebellar pathway in kittens. Brain Res. 316, 247–254. 10.1016/0165-3806(84)90309-2 6467015

[B44] Pearlstein E. Bras H. Deneris E. S. Vinay L. (2011). Contribution of 5-HT to locomotion—the paradox of *Pet-1*^−/−^ mice. Eur. J. Neurosci. 33, 1812–1822. 10.1111/j.1460-9568.2011.07679.x 21501257

[B45] Repapi E. Sayers I. Wain L. V. Burton P. R. Johnson T. Obeidat M.. (2009). Genome-wide association study identifies five loci associated with lung function. Nat. Genet. 42, 36–44. 10.1038/ng.501 20010834 PMC2862965

[B46] Rochefort C. Arabo A. Andre M. Poucet B. Save E. Rondi-Reig L. (2011). Cerebellum shapes hippocampal spatial code. Science 334, 385–389. 10.1126/science.1207403 22021859

[B47] Rochefort C. Lefort J. M. Rondi-Reig L. (2013). The cerebellum: a new key structure in the navigation system. Front. Neural Circuits 7:35. 10.3389/fncir.2013.00035 23493515 PMC3595517

[B48] Schmahmann J. D. Pandya D. N. (1991). Projections to the basis pontis from the superior temporal sulcus and superior temporal region in the rhesus monkey. J. Comp. Neurol. 308, 224–248. 10.1002/cne.903080209 1716269

[B49] Smith P. F. Zheng Y. Horii A. Darlington C. L. (2005). Does vestibular damage cause cognitive dysfunction in humans? J. Vestib. Res. 15, 1–9. 10.3233/VES-190672 15908735

[B50] Sripanidkulchai K. Wyss J. M. (1986). Thalamic projections to retrosplenial cortex in the rat. J. Comp. Neurol. 254, 143–165. 10.1002/cne.902540202 3794004

[B51] Sun Y. Nguyen A. Q. Nguyen J. P. Le L. Saur D. Choi J.. (2014). Cell-type-specific circuit connectivity of hippocampal CA1 revealed through Cre-dependent rabies tracing. Cell Rep. 7, 269–280. 10.1016/j.celrep.2014.02.030 24656815 PMC3998524

[B52] Timmann D. Daum I. (2007). Cerebellar contributions to cognitive functions: a progress report after two decades of research. Cerebellum 6, 159–162. 10.1080/14734220701496448 17786810

[B53] Timmann D. Drepper J. Frings M. Maschke M. Richter S. Gerwig M.. (2010). The human cerebellum contributes to motor, emotional, and cognitive associative learning. A review. Cortex 46, 845–857. 10.1016/j.cortex.2009.06.009 19665115

[B54] Tolbert D. L. (1989a). Absence of impulse activity in cortical neurons with transient projections to the cerebellum. Dev. Brain Res. 50, 241–249. 10.1016/0165-3806(89)90200-9 2611987

[B55] Tolbert D. L. (1989b). Somatotopically organized transient projections from the primary somatosensory cortex to the cerebellar cortex. Dev. Brain Res. 45, 113–127. 10.1016/0165-3806(89)90013-8 2917405

[B56] Tolbert D. L. Panneton W. M. (1983). Transient cerebrocerebellar projections in kittens: postnatal development and topography. J. Comp. Neurol. 221, 216–228. 10.1002/cne.902210209 6655083

[B57] Tolbert D. L. Panneton W. M. (1984). The transience of cerebrocerebellar projections is due to selective elimination of axon collaterals and not neuronal death. Brain Res. 318, 301–306. 10.1016/0165-3806(84)90034-8 6208975

[B58] Ugolini G. (1995). Specificity of rabies virus as a transneuronal tracer of motor networks: transfer from hypoglossal motoneurons to connected second-order and higher order central nervous system cell groups. J. Comp. Neurol. 356, 457–480. 10.1002/cne.903560312 7642806

[B59] van Groen T. Wyss J. M. (1990). Connections of the retrosplenial granular a cortex in the rat. J. Comp. Neurol. 300, 593–606. 10.1002/cne.903000412 2273095

[B60] van Groen T. Wyss J. M. (2003). Connections of the retrosplenial granular b cortex in the rat. J. Comp. Neurol. 463, 249–263. 10.1002/cne.10757 12820159

[B61] van Groen T. Kadish I. Wyss J. M. (2002a). Old rats remember old tricks; memories of the water maze persist for 12 months. Behav. Brain Res. 136, 247–255. 10.1016/s0166-4328(02)00137-7 12385811

[B62] van Groen T. Kadish I. Wyss J. M. (2002b). The role of the laterodorsal nucleus of the thalamus in spatial learning and memory in the rat. Behav. Brain Res. 136, 329–337. 10.1016/s0166-4328(02)00199-7 12429394

[B63] van Groen T. Kadish I. Wyss J. M. (2002c). Species differences in the projections from the entorhinal cortex to the hippocampus. Brain Res. Bull. 57, 553–556. 10.1016/s0361-9230(01)00683-9 11923027

[B64] Vann S. D. Aggleton J. P. Maguire E. A. (2009). What does the retrosplenial cortex do? Nat. Rev. Neurosci. 10, 792–802. 10.1038/nrn2733 19812579

[B65] Vertes R. P. McKenna J. T. (2000). Collateral projections from the supramammillary nucleus to the medial septum and hippocampus. Synapse 38, 281–293. 10.1002/1098-2396(20001201)38:3<281::aid-syn7>3.0.co;2-6 11020231

[B66] Watson T. C. Obiang P. Torres-Herraez A. Watilliaux A. Coulon P. Rochefort C.. (2019). Anatomical and physiological foundations of cerebello-hippocampal interaction. eLife 8:e41896. 10.7554/eLife.41896 31205000 PMC6579515

[B67] Whitney M. S. Shemery A. M. Yaw A. M. Donovan L. J. Glass J. D. Deneris E. S. (2016). Adult brain serotonin deficiency causes hyperactivity, circadian disruption, and elimination of siestas. J. Neurosci. 36, 9828–9842. 10.1523/JNEUROSCI.1469-16.2016 27656022 PMC5030349

[B68] Wickersham I. R. Finke S. Conzelmann K. K. Callaway E. M. (2007a). Retrograde neuronal tracing with a deletion-mutant rabies virus. Nat. Methods 4, 47–49. 10.1038/nmeth999 17179932 PMC2755236

[B69] Wickersham I. R. Lyon D. C. Barnard R. J. Mori T. Finke S. Conzelmann K. K.. (2007b). Monosynaptic restriction of transsynaptic tracing from single, genetically targeted neurons. Neuron 53, 639–647. 10.1016/j.neuron.2007.01.033 17329205 PMC2629495

[B70] Wild J. M. Williams M. N. (2000). A direct cerebrocerebellar projection in adult birds and rats. Neuroscience 96, 333–339. 10.1016/s0306-4522(99)00546-1 10683573

[B71] Wyss J. M. Van Groen T. (1992). Connections between the retrosplenial cortex and the hippocampal formation in the rat: a review. Hippocampus 2, 1–11. 10.1002/hipo.450020102 1308170

[B73] Xiao X. Li J. McCown T. J. Samulski R. J. (1997). Gene transfer by adeno-associated virus vectors into the central nervous system. Exp. Neurol. 144, 113–124. 10.1006/exnr.1996.6396 9126160

[B72] Xiao X. Li J. Samulski R. J. (1996). Efficient long-term gene transfer into muscle tissue of immunocompetent mice by adeno-associated virus vector. J. Virol. 70, 8098–8108. 8892935 10.1128/jvi.70.11.8098-8108.1996 PMC190884

