# Supplementary figures and images for: A New Projection From the Deep Cerebellar Nuclei to the Hippocampus via the Ventrolateral and Laterodorsal Thalamus in Mice
Source: Front Neural Circuits. 2019 Aug 9;13:51. doi: 10.3389/fncir.2019.00051 (PMC6695568; doi:10.3389/fncir.2019.00051)

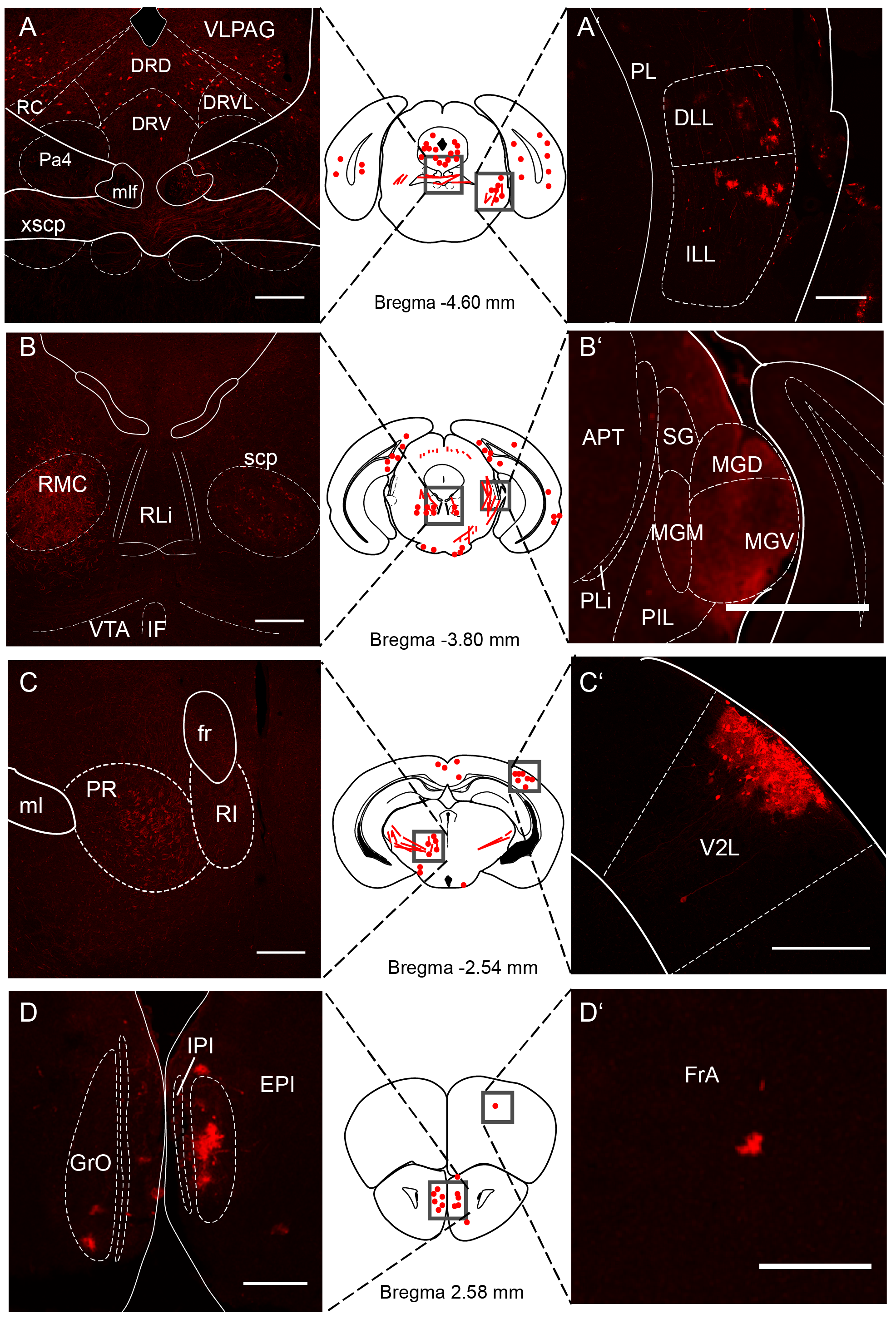

Supplement: FIGURE S1 — Additional polysynaptic targets observed after injection of the polysynaptic anterograde tracer AAV8-CMV-WGA-Cre in the DCN of tdTomato+ mice. (A) Example single tdTomato+ cell bodies were imaged from the dorsal raphe nuclei [dorsal part (DRD), ventral (DRV), ventrolateral (DRVL), the raphe cap (RC) and ventrolateral periaqueducal gray (VLPAG)] and the lateral lemniscus, dorsal (DLL) and intermediate nucleus (ILL; A’) at −4.60 mm from Bregma. Scale bars: 250 μm. (B) Example single tdTomato+ cell bodies were imaged from the magnocellular red nucleus (RMC) and superior cerebellar peduncle (scp) and right dorsal and ventral medial geniculate nucleus (MGD, MGV; B’) at −3.80 mm from Bregma. tdTomato+ neurites were additionally seen in the posterior intralaminar thalamic nucleus (PIL), suprageniculate thalamic nucleus (SG) and the medial part of the medial geniculate nucleus (MGM). Scale bars: 250 μm (C) Example single tdTomato+ cell bodies imaged from the prerubral field (PRl; C) and lateral secondary visual cortex (V2L; C’) at −2.54 mm from Bregma. Scale bars: 250 μm. (D) Polysynaptically tdTomato+ cells at 2.58 mm from Bregma seen in the granular cell layer of the olfactory bulb (GrO) and internal plexiform layer of the olfactory bulb (IPI) and frontal association cortex (FrA; D’) Scale bars: 250 μm. The mouse brains in this figure has been reproduced from Franklin and Paxinos (2001). [file Image_1.TIF]

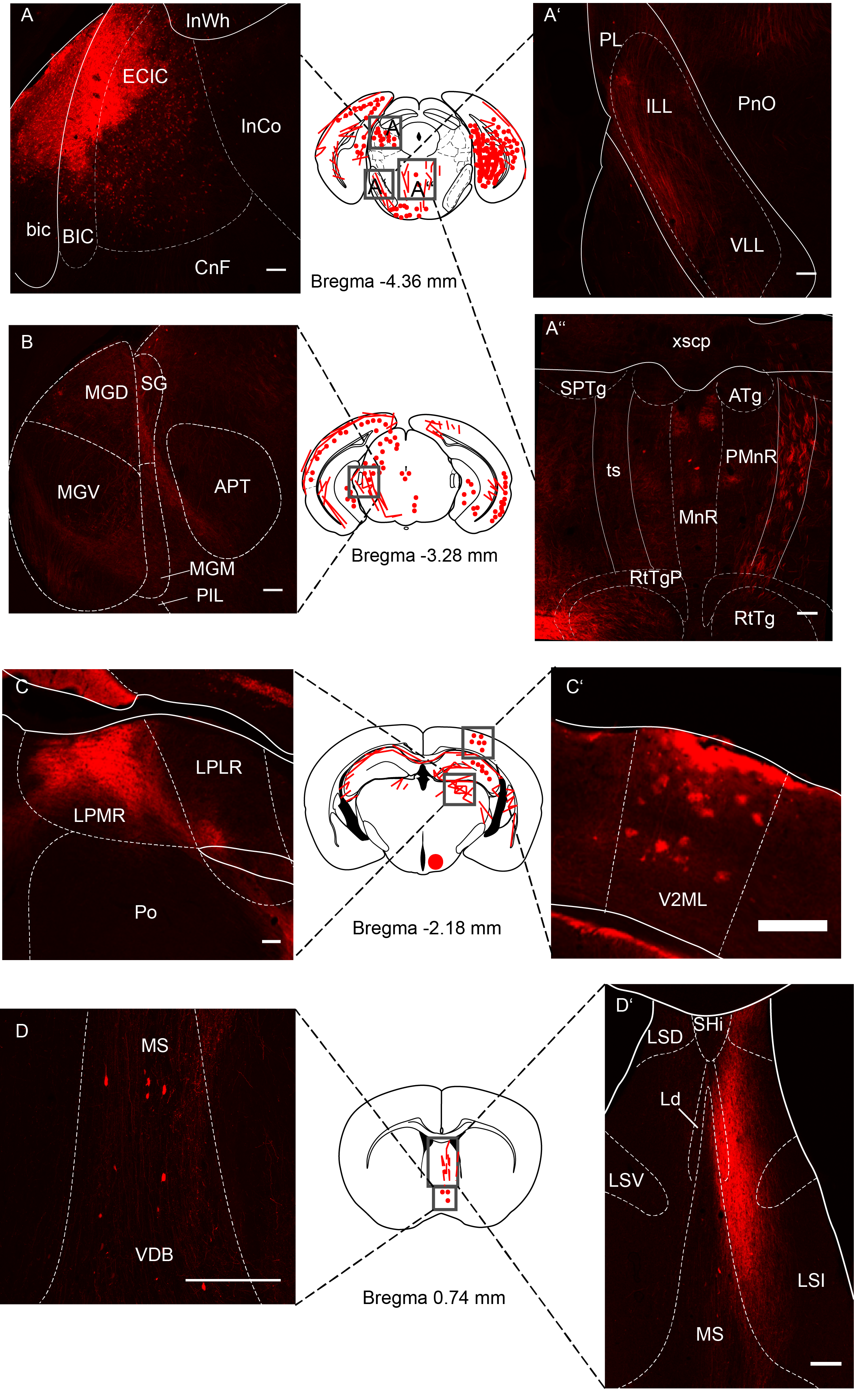

Supplement: FIGURE S2 — Additional polysynaptic targets observed after injection of the polysynaptic anterograde tracer AAV8-CMV-WGA-Cre in the dentate gyrus of tdTomato+ mice. (A) Polysynaptic tdTomato+ cells at −4.36 mm from Bregma in the external cortex of the inferior colliculus (ECIC), nucleus of the brachium of the inferior colliculus (BIC) and the brachium colliculus (bic). (A’) tdTomato+ neurites in the lateral lemniscus, intermediate (ILL) and ventral nuclei (VLL). Scale bar: 100 μm. (A”) tdTomato+ cell bodies and neurites in the tectospinal tract (ts), median raphe nucleus (MnR) and paramedian raphe nucleus (PMnR) and the reticulotegmental nucleus of the pons (RtTg). Scale bars: 100 μm. (B) Exemplary tdTomato+ neurites in the medial geniculate nucleus, dorsal (MGD), ventral (MGV) and medial (MGM) parts and the suprageniculate thalamic nucleus (SG) at −3.28 mm from Bregma. Scale bar: 100 μm (C) Exemplary tdTomato+ neurites in the lateral posterior thalamic nucleus, mediorostral (LPMR) and laterorostral parts (LPLR). (C’) tdTomato+ cell bodies in the secondary visual cortex, mediolateral area (V2ML). Scale bars 100 μm. (D) At 0.74 mm from Bregma, tdTomato+ cell bodies were seen in the medial septal nucleus (MS) and in the nucleus of the vertical limb of the diagonal band (VDB). Scale bar: 250 μm. (D’) Intense tdTomato+ neurites in the lambdoid septal zone (Ld) and dorsal (LSD) and intermediate nuclei of the lateral septal nucleus (LSI). Scale bars: 250 μm. The mouse brains in this figure has been reproduced from Franklin and Paxinos (2001). [file Image_2.TIF]

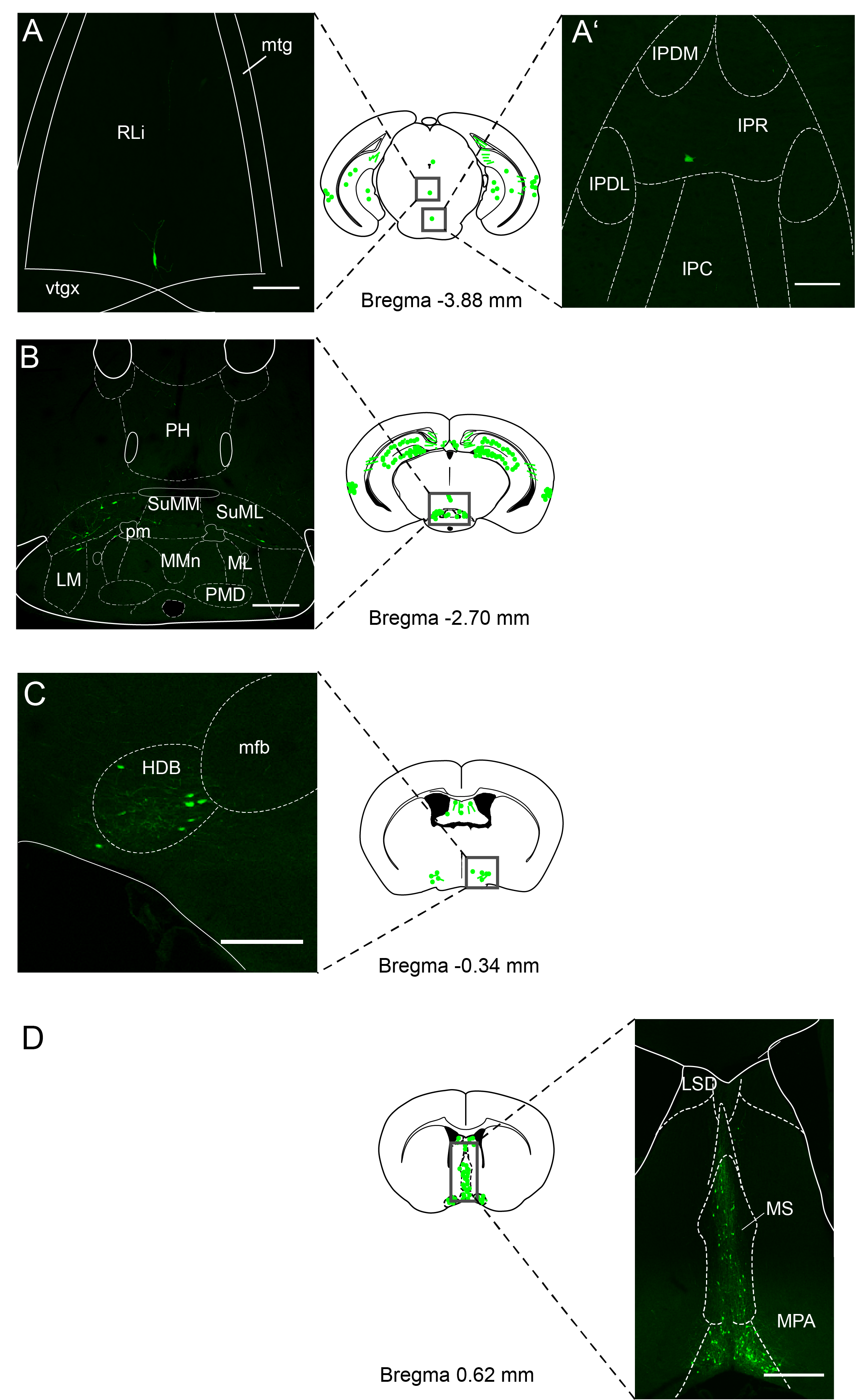

Supplement: FIGURE S3 — Additional monosynaptic connected areas observed after injection of the retrograde tracer SADΔG-eGFP in the Hippocampus of C57/Bl6 mice. (A) Coronal brain section at −3.88 mm from Bregma depicting an eGFP+ neuron (green dots) in the rostral linear nucleus of the raphe (RLi) and interpeduncular nucleus, rostral subnucleus (IPR, A’) by retrograde monosynaptic transport from the hippocampus. Scale bar: 100 μm. (B) Confocal image from boxed area depicting eGFP+ cell bodies in the medial and lateral supramammillary nucleus (SuML, SuMM) at −2.70 mm from Bregma. Scale bar: 500 μm. (C) Confocal image of eGFP+ neurons in the nucleus of the horizontal limb of the diagonal band (HDB) at – 0.34 mm from Bregma. Scale bar: 200 μm. (D) Confocal image of a multiple eGFP+ cell bodies in the medial septal nucleus (MS) and medial preoptic area (MPA). Scale bar: 250 μm. The mouse brains in this figure has been reproduced from Franklin and Paxinos (2001). [file Image_3.TIF]

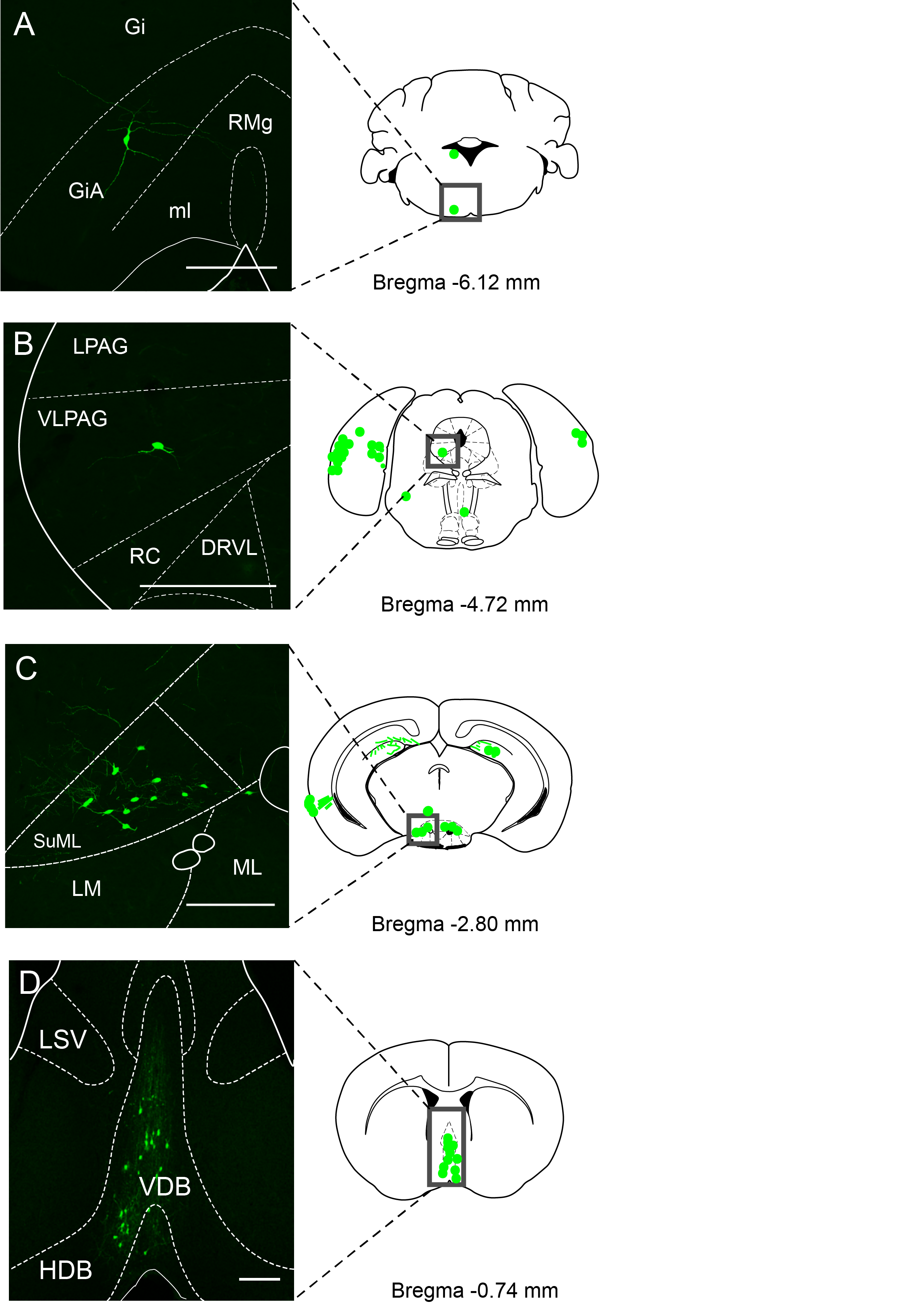

Supplement: FIGURE S4 — Additional monosynaptic connected areas observed after injection of the retrograde tracer SADΔG-eGFP in the Dentate Gyrus of C57/Bl6 mice. (A) Coronal brain section at −6.12 mm from Bregma depicting a eGFP+ neuron (green dots) in the alpha part of the gigantocellular reticular nucleus (GiA) by retrograde monosynaptic transport from the dentate gyrus (DG). Scale bar: 250 μm. (B) Confocal image from boxed area depicting a single eGFP+ cell body in the ventrolateral periaqueductal gray (VLPAG) at −4.72 mm from Bregma. Scale bar: 250 μm. (C) Confocal image of eGFP+ neurons in the lateral supramammillary nucleus (SuML) at −2.80 mm from Bregma. Scale bar: 250 μm. (D) Confocal image of a multiple eGFP+ cell bodies in the nucleus of the ventral limb of the diagonal band (VDB). Scale bar: 250 μm. The mouse brains in this figure has been reproduced from Franklin and Paxinos (2001). [file Image_4.TIF]

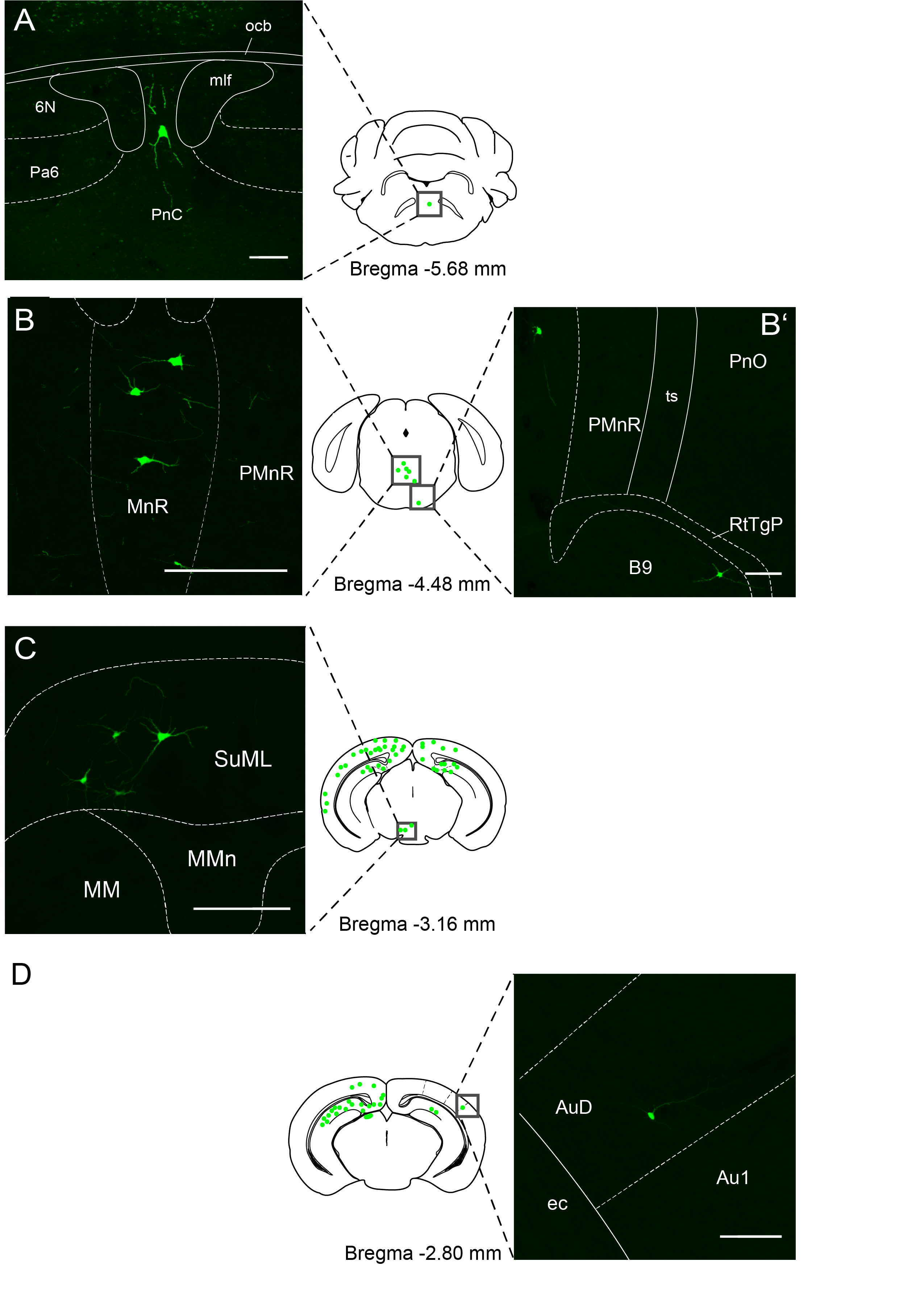

Supplement: FIGURE S5 — Additional monosynaptic connected areas observed after injection of the retrograde tracer SADΔG-eGFP in the retrosplenial cortex of C57/Bl6 mice. (A) Coronal brain section at −5.68 mm from Bregma depicting a eGFP+ neuron (green dots) in the pontine reticular nucleus (PnC) by retrograde monosynaptic transport from the retrosplenial cortex (RC). Scale bar: 100 μm. (B,B’) Confocal images from boxed areas depicting eGFP+ cell bodies in the medial raphe nucleus (MnR; B, Scale bar: 250 μm) and B9 serotonergic cells (B’, Scale bar: 100 μm) at −4.48 mm from Bregma. (C) Confocal image of eGFP+ neurons in the lateral supramammillary nucleus (SuML) at −3.16 mm from Bregma. Scale bar: 250 μm. (D) Confocal image of a single rabies-infected cell in the secondary auditory cortex (AuD). Scale bar: 100 μm. The mouse brains in this figure has been reproduced from Franklin and Paxinos (2001). [file Image_5.TIF]

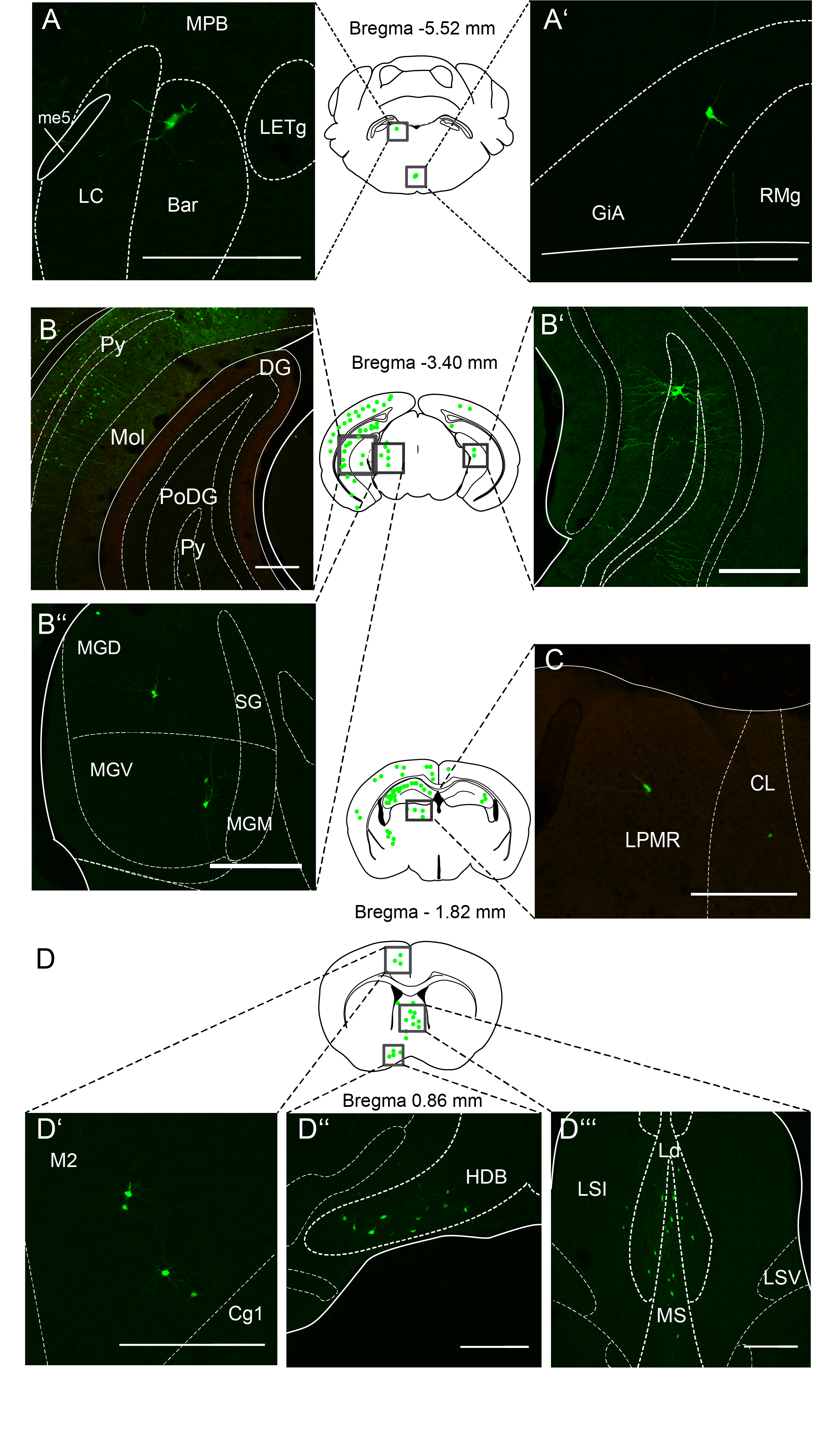

Supplement: FIGURE S6 — Additional monosynaptic connected areas observed after injection of the retrograde tracer SADΔG-eGFP in the rhinal cortex of C57/Bl6 mice. (A) Confocal image showing higher magnification of a eGFP+ neuron in the Barrington’s nucleus (Bar) in the pons at −5.52 mm from Bregma. Scale bar: 250 μm (A’) Single eGFP+ cell in the alpha part of the gigantocellular reticular nucleus (GiA) in the pons at −5.52 mm from Bregma. Scale bar: 250 μm. (B) Several pyramidal cell-like neurons in the CA1 region and pyramidal cell layer (Py) at −3.80 mm from Bregma on the ipsilateral side. Scale bar: 250 μm. (B’) Only one cell in the Py was seen on the contralateral side. Scale bar: 250 μm. (B”) Confocal image showing several eGFP+ cells in the dorsal (MGD) and ventral (MGV) parts of medial geniculate nucleus at −3.40 mm from Bregma. Scale bar: scale bar: 250 μm. (C) Brain scheme at −1.82 mm. Green dots represent eGFP+ cells in all analyzed mice in the RSC, ipsilateral CA1 and CA2. High magnification image showing one representative single eGFP+ neuron found in the ipsilateral mediorostral lateral posterior (LPMR) of the thalamus. Scale bar: 250 μm (D) Brain scheme at 0.86 mm from Bregma showing representative summarized eGFP+ cells. Squared boxes represent areas of confocal images of the ipsilateral secondary motor cortex (M2, D’), ipsilateral nucleus of the horizontal limb (HDB, D”) and medial septal nucleus (MS) and lambdoid zone (Ld; D”’). Scale bars: 250 μm. The mouse brains in this figure has been reproduced from Franklin and Paxinos (2001). [file Image_6.TIF]

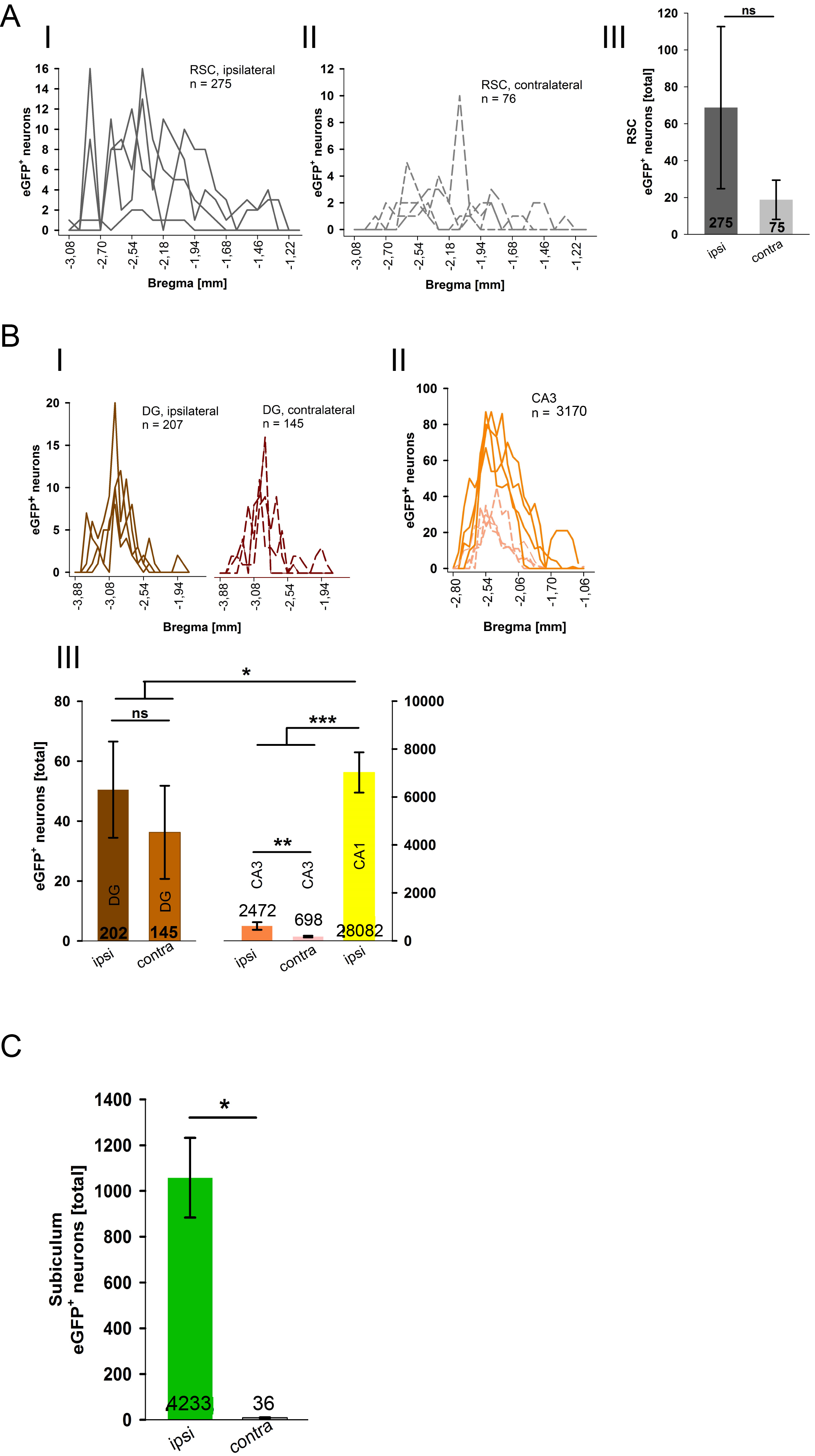

Supplement: FIGURE S7 — Additional statistical analysis of all eGFP+ neurons observed in different regions on the murine brain after injection of the retrograde tracer SADΔG-eGFP in the rhinal cortex of C57/Bl6 mice. (A) eGFP+ cells that were found in the RSC at different mm from Bregma. The four traces represent the analyzed n = 4 mice. (AI) A total of 275 eGFP+ cells were found in the ipsilateral RSC, while only 76 cells were found in the contralateral RSC (AII). (AIII) No statistical difference was found between ipsi., and contralateral sites. T-test, p = 0.069. (B) Summary of eGFP+ cells that were found in the DG and CA3 at different mm from Bregma. The four traces represent the analyzed n = 4 mice. (BI) A total of 207 eGFP+ cells were found in the ipsilateral DG, while 145 cells were found in the contralateral DG. No statistical difference was found between sites. T-test, p = 0.249 (BIII). (BII) Traces of all eGFP+ cells observed in the ipsilateral (orange thick lines, 2,472 cells) and contralateral (dashed lines, 698) CA3 region of the hippocampus. Three-thousand one-hundred and seventy cells were found in all analyzed mice, with significantly more input on the RC from the ipsilateral CA3 cells (t-test, p = 0.002; BIII). (BIII) A total of 28,082 eGFP+ cells were found in the CA1, with only eight cells found on the contralateral site (data not shown). Significantly more cells from the CA1 region are connected to the RC when compared to all CA3 cells (t-test, p = ≤ 0.001) and all cells from the DG (t-test, p = 0.029). (C) Total eGFP+ neurons observed in the subiculum. Significantly more cells from the ipsilateral S (4,233) compared to the contralateral S (36) provide input to the RC (t-test, p = 0.029). Significance for comparisons: ns not significant; *p ≤ 0.05; **p ≤ 0.01; ***p ≤ 0.001. [file Image_7.TIF]
